# Evaluation of sNfL as a Biomarker for Paclitaxel-Induced Peripheral Neurotoxicity Through an Integrated PKPD Model

**DOI:** 10.1007/s11095-026-04053-z

**Published:** 2026-03-13

**Authors:** Eman I. K. Ibrahim, Milda Girdenyté, Yang Hu, Lorenzo Di Cesare Mannelli, David Balayssac, Jérôme Busserolles, Diethilde Theil, Gautier Roussignol, Olivier Perrault, Nathalie Le Berre, Franck Chanut, Mohamed Slaoui, Irena Loryan, Lena E. Friberg

**Affiliations:** 1 Department of Pharmacy, Uppsala University, Uppsala, Sweden; 2 Pharmacy and Pharmacology Center, Institute of Biomedical Sciences, Faculty of Medicine, Vilnius University, Vilnius, Lithuania; 3 Non-Clinical Safety & DMPK, BioNTech SE, Mainz, Germany; 4 Department of Neuroscience, Psychology, Drug Research and Child Health – Neurofarba – Section of Pharmacology and Toxicology, University of Florence, Florence, Italy; 5 Direction de La Recherche Clinique Et de L’Innovation, Université Clermont Auvergne, INSERM, NEURO-DOL, CHU Clermont-Ferrand, Clermont-Ferrand, U1107 France; 6 Université Clermont Auvergne, INSERM, NEURO-DOL, Clermont-Ferrand, U1107 France; 7 Novartis Institutes for Biomedical Research, Cambridge, MA USA; 8 Roche Pharmaceutical Research and Early Development , Roche Innovation Center, Basel, Switzerland; 9 Preclinical Safety, Sanofi, R&D, Montpellier, France; 10 Preclinical Safety, Sanofi, R&D, Chilly Mazarin & Vitry, France; 11 Preclinical Safety, Sanofi, R&D, Vitry, France

**Keywords:** brain, dorsal root ganglia, modelling & simulation, paclitaxel-induced peripheral neurotoxicity (PIPN), peripheral nerves, pharmacokinetic-pharmacodynamic (PKPD) model, PIPN sites, serum neurofilament light chain (sNfL), tubulin binding, unbound (free) paclitaxel

## Abstract

**Background:**

Serum neurofilament light chain (sNfL), a biomarker of axonal damage, has shown promise in clinical studies for monitoring paclitaxel-induced peripheral neurotoxicity (PIPN). The latter involves pathological changes in PIPN sites such as the dorsal root ganglia, peripheral nerves, and brain. However, the mechanistic link between paclitaxel and NfL concentrations in these tissues remains poorly understood, necessitating preclinical investigation.

**Methods:**

We developed a semi-mechanistic pharmacokinetic-pharmacodynamic model to characterize: (i) total and unbound paclitaxel concentrations in plasma, as well as in extracellular and intracellular compartments of PIPN sites; (ii) paclitaxel–tubulin complex formation; and (iii) NfL kinetics. The model was built using de novo-generated and previously reported data from rodents.

**Results:**

Plasma pharmacokinetics of paclitaxel was captured using a two-compartment model, including Cremophor EL trapping and nonlinear tissue distribution. Paclitaxel pharmacokinetics in PIPN sites incorporated paclitaxel transport across the blood-to-PIPN sites barriers and paclitaxel–tubulin binding, described by capacity-limited kinetics with increased tubulin binding upon repeated plasma exposure. NfL kinetics in serum and cerebrospinal fluid were described using turnover models, with NfL leakage driven by paclitaxel–tubulin complex formation in PIPN sites. The model robustly predicted paclitaxel exposure across multiple doses and studies. While NfL predictions aligned with single-dose data, the model slightly underpredicted sNfL levels in an external validation dataset after repeated dosing of paclitaxel at 15 mg/kg, suggesting additional mechanisms may be involved.

**Conclusions:**

Overall, the model successfully described the relationship between paclitaxel exposure and sNfL kinetics, offering a model-based framework for translational studies.

**Supplementary Information:**

The online version contains supplementary material available at 10.1007/s11095-026-04053-z.

## Introduction

Paclitaxel (PTX) is an anticancer drug that is commonly used for non-small cell lung, breast, and ovarian cancers, and Kaposi sarcoma. In rapidly proliferating tumor cells, PTX binds to microtubules, stabilizes their structure, and enhances their polymerization, resulting in cell cycle arrest [1]. Although neurons are non-dividing cells, they remain susceptible to PTX, likely due to PTX–tubulin binding that disrupts axonal transport and destabilizes neuronal architecture, ultimately leading to axonal degeneration and contributing to its neurotoxic effects [1]. PTX-induced peripheral neurotoxicity (PIPN) is one of the major dose-limiting and long-lasting side effects of PTX treatment [2]. Two-thirds of patients experience PIPN-related symptoms [2, 3]. The lack of effective preventive and curative treatments [3] is partly related to an incomplete understanding of the pharmacokinetics-pharmacodynamics (PKPD) of PTX in the key PIPN sites, which is essential for evaluating and monitoring PIPN development.

Multiple molecular and cellular mechanisms of PIPN, including microtubule disruption and axonal transport impairment, are linked to specific anatomical sites in the peripheral nervous system (PNS), i.e., dorsal root ganglia (DRG) and peripheral nerves (PN) [1]. Additionally, it is hypothesized that the central nervous system (CNS) has an indirect role in PIPN development due to brain and spinal cord sensitization and compensation mechanisms caused by altered PNS activity [4]. Due to their well-documented contribution to PIPN pathology, these tissues (i.e., DRG, PN, and brain) are, herein, collectively termed PIPN sites.

PTX distribution to PIPN sites has been studied in rodents [5–12], including a detailed characterization of the extent of unbound and total PTX transport across biological barriers such as blood-DRG (BDB), blood-nerve (BNB), and blood–brain (BBB) barriers [7, 8]. PTX accumulation in PIPN sites has been observed with repeated dosing, leading to behavioral changes due to nociceptive disorders in rodents [11–15]. However, such distributional studies are not feasible in clinical settings, highlighting the need for a translational, model-based, mechanistic framework to link preclinical findings to patients. Several PK models, empirical and mechanism-based ones, have been developed to characterize the plasma PK of PTX in humans and describe the differences in PK between the formulations [16–19]. Preclinical studies have demonstrated that PK profiles in PIPN sites are not parallel to those in plasma [5–8], underlying the limitations of PTX plasma exposure-based characterization of PIPN development [5, 6]. Hence, a plasma-PIPN site PK model could enhance our understanding of the relationship between systemic and tissue PTX concentrations and enable more reliable extrapolation of preclinical data to humans.

Several liquid biomarkers were studied for monitoring chemotherapy-induced peripheral neuropathy [13, 20], with serum neurofilament light chain (sNfL) shown to be a promising biomarker for PIPN [11–13, 15, 21–23]. NfL is part of the cytoskeleton, and together with other neurofilaments and microtubules, supports neuronal structure [24]. NfL leakage into the cerebrospinal fluid (CSF) and serum has been correlated to axonal damage in the CNS and has been established as a monitoring biomarker for several CNS disorders [25]. In parallel, various mathematical modeling approaches were employed to characterize NfL kinetics in healthy and neurodegenerative conditions [26–29].

Clinical evidence also suggests that elevated sNfL levels are associated with increased severity of PIPN [21–23, 30]. Similarly, several rodent studies have demonstrated that repeated PTX dosing increased sNfL levels in a dose-dependent manner, concomitant with histopathological changes in PNS [11–13, 15]. In addition, *in vitro* studies by Huehnchen *et al* showed that NfL release from induced pluripotent stem cell–derived sensory neurons exposed to PTX is dose- and time-dependent [21]. Yet, the relationship between PTX and NfL either in plasma or PIPN sites has not been investigated preclinically or clinically. In this regard, the availability of data on NfL concentrations in plasma and PIPN sites, both at baseline levels and after PTX administration, is a limiting factor. Given the heterogeneous distribution of NfL in nervous tissues, the extent to which the individual PIPN sites may contribute to increased sNfL levels after PTX administration remains unclear. Lack of data and knowledge gaps impede a mechanistic understanding of how NfL levels in the CNS and PNS contribute to sNfL kinetics following PTX administration. In this context, PKPD modeling offers a powerful, quantitative approach to link PTX exposure at PIPN-relevant sites with NfL kinetics, providing mechanistic insight into neurotoxicity progression and enabling the integration of data from diverse experimental sources. The overarching goal of this study was to establish a translational, experimental, and model-based framework, with the ultimate goal of supporting individualized treatment strategies and evaluating effective prevention and therapeutic interventions. We aimed to characterize concentration–time profiles of PTX in plasma, DRG, PN, and brain, along with NfL kinetics after PTX administration using a nonlinear mixed-effect modeling approach. The model was developed using de novo generated* in vivo* data on longitudinal NfL data in serum, CSF, and PIPN sites after a single dose of PTX formulated in Cremophor EL with ethanol (CrEL-PTX). The model performance was validated using external previously reported *in vivo* data on PTX and sNfL concentrations in mice and rats [5–8, 11, 12].

## Methods

### NfL Dataset Generation

#### Animals

The evaluation of NfL kinetics in serum, CSF, and PIPN sites after a single CrEL-PTX dose has been done by performing a 10-day longitudinal study. In total, sixteen 8–12 week-old male Sprague–Dawley rats (Taconic, Lille Skensved, Denmark) were used for the *in vivo* experiments. After arrival, the animals were housed in groups under 20 to 22 °C and 40 to 50% humidity, in a 12-h light/dark cycle with ad *libitum* food and water. All animals were acclimatized for one week before the experiment. Experiments were performed following guidelines from the Swedish National Board for Laboratory Animals, approved by the Animal Ethics Committee of Uppsala, Sweden (Ethical Approval Dnr 5.8.18–09258/2024). The performed studies were not randomized or blinded for the treatment arms.

#### Study Design and Bioanalysis

The study design in terms of PTX dose and sampling time was optimized in a pilot study. Two dose levels of CrEL-PTX at 10 mg/kg and 15 mg/kg were selected in the pilot study, based on a previously reported link to PIPN development [11]. In our hands, intravenous bolus administration of 15 mg/kg CrEL-PTX was associated with 50% mortality. Hence, we selected 10 mg/kg for the longitudinal study, where all animals were injected intravenously either with saline (control, *N* = 3) or 10 mg/kg bolus CrEL-PTX (*N* = 7) via the tail vein. The injection solution of 4 mg/mL of CrEL-PTX was prepared *ex tempore* by diluting 6 mg/mL of PTX injection solution with saline, according to the manufacturer’s instructions (Paclitaxel Fresenius Kabi, Uppsala, Sweden). Pain and distress signs were evaluated in animals according to Uppsala University guidelines.

Serum sampling was performed at time 0 (pre-PTX dose) and at 24, 48, 72, 96, 168, 192, 216, and 240 h post-PTX dose in the morning. To collect serum samples from the right leg vein, animals were heated under an infrared lamp for 10–20 min, and blood samples (∼180 µL) were collected into low-binding microtubes. The blood was allowed to clot for 15–20 min followed by centrifugation at 10,000 rpm for 5 min at room temperature using VWR® Micro Star 12 microcentrifuge (VWR, Stockholm, Sweden). Serum samples were collected and stored at −80°C. After 72 (*N* = 3) and 240 (*N* = 4) hours post-PTX administration, animals were anesthetized by inhalation of 2.5% isoflurane (Abbot Scandinavia, Solna, Sweden) balanced with 3 L/min oxygen. CSF was collected from the *cisterna magna.* To evaluate the impact of anesthesia and surgical interventions required to get access to the *cisterna magna* on sNfL levels, an additional serum sample was collected via heart puncture after the collection of CSF. Hippocampus, DRG, and PN, represented by sciatic nerve (SN) were dissected and collected in pre-weighed 0.5 mL pre-filled bead tubes (VWR® Soft Tissue Homogenizing Mix, 1.4 mm Ceramic Beads; VWR, Stockholm, Sweden). All samples were weighed and kept on dry ice, followed by storage at −80°C pending bioanalysis.

Prior to bioanalysis, tissue samples were homogenized in RIPA buffer (Thermo Fisher Scientific Pierce Biotechnology, Rockford, IL, US) with protease inhibitors (cOmplete™, Mini Protease Inhibitor Cocktail, Roche Diagnostics GmbH, Germany) according to the manufacturer’s instructions. Four (hippocampus and SN) and nine (DRG) volumes of RIPA buffer per tissue weight were added into tubes. The tissues were homogenized using a 4-Place Mini Bead Mill Homogenizer (VWR, Stockholm, Sweden) for 2 min (hippocampus and DRG) or 6 min (SN). After mechanical homogenization, samples were centrifuged at 14,800 rpm for 20 min at 4 °C using VWR® Micro Star 21R microcentrifuge (VWR, Stockholm, Sweden). Fifty µL of supernatant was collected in a polypropylene Corning 0.5 mL V-bottom 96-well plate (VWR, Stockholm, Sweden) and stored at −80°C pending bioanalysis.

The NfL concentration in serum, CSF, hippocampus, DRG, and SN samples was analyzed simultaneously using the Simple Plex Rat NF-L Cartridge (Kit ID: 360,235, ProteinSimple, Minneapolis, US) with Ella™ Automated ELISA. On the day of bioanalysis, all samples were further diluted using the sample diluent. Serum and CSF samples were diluted according to the manufacturer’s instructions, i.e., 1:1 (v:v). For the NfL concentration analysis in the tissues, a dilution factor was optimized to 1,000 for the hippocampus and 800,000 for the DRG and SN samples. The calibration range was 2.7–10,290 pg/mL.

#### PTX Pharmacokinetic Dataset Collection

PK data were obtained from six different sites/sources summarized in Table I: 1) Uppsala University (UU) [7, 8], 2) University of Florence (UNIFI, Italy), 3) University Clermont Auvergne (UCA, France), 4) Sanofi laboratories (SARD, France) [11, 12], and digitized data from 5) Nakamura, I.* et al*. [6] and 6) from Wozniak, K. M. *et al*. [5]. Total PTX concentration in plasma and nervous tissues (DRG, SN, and brain) was determined at various time points after intravenous administration of different doses of PTX to mice and rats.

**Table I:** Summary of Paclitaxel (PTX) Pharmacokinetic (PK) and Serum Neurofilament Light Chain (sNfL) Data From *in vivo* Studies Used to Build an Integrated PTX Plasma–PIPN–Sites PK Model and to Validate the Performance of the PTX PK-NfL Model

Data source	Rodent type	Number of animals	Dosing regimen	Type of samples collected for PTX measurement	Serum/plasma collection for NfL measurement
1- Uppsala University (UU)	Wistar Hanover rats	3	2.6 mg/kg, IV, single dose	Plasma, SN, DRG, BRN	Serum
		4	5 mg/kg, single dose (4 h IV infusion)		
	Sprague Dawley rats	3	2.6 mg/kg, IV, single dose		
2- University of Florence (UNIFI)	Wistar Hanover rats	6	5 mg/kg, IV, QW for 2 weeks	Plasma, SN, DRG	Plasma
		6	5 mg/kg, IV, QW for 4 weeks		
3- University Clermont Auvergne (UCA)	Wistar Hanover rats	8	5 mg/kg, IV, QW for 2 weeks	Plasma, SN, DRG, BRN	Plasma
		12	5 mg/kg, IV, QW for 4 weeks		
4- Sanofi laboratories (SARD)	CD1 mice	5	5 mg/kg, IV, TIW at week 1 and QIW at week 2	Plasma, SN, DRG, BRN	Plasma
		5	10 mg/kg, IV, TIW at week 1 and QIW at week 2		
		5	15 mg/kg, IV, TIW at week 1 and QIW at week 2		
5- Nakamura, I. *et al*. 2016 [6]	Sprague Dawley rats	(-)*	7.5 mg/kg, IV, QW for 6 weeks	Plasma, DRG, BRN	No
			7.5 mg/kg, IV, single dose		
6-Wozniak, K. M. *et al*. 2017 [5]	BALB/c mice	(-)*	30 mg/kg, IV, Q2Dx3 for 2 weeks	Plasma, SN, DRG	No
			30 mg/kg, IV, single dose		

*Data obtained from published figures by digitizing the mean values at each time-point using an online tool (https://automeris.io/WebPlotDigitizer/).* PIPN*—paclitaxel-induced peripheral neurotoxicity,* QW* – every week, *QIW* – four times a week, *TIW* – three times a week,* Q2Dx*3 – every 2 days for 3 doses, *IV* – intravenous, *SN* – sciatic nerve, *DRG* – dorsal root ganglia, *BRN* – brain

#### An Integrated PTX PK-NfL Model Building

The PTX PK-NfL model building was performed in four sequential steps: 1) developing a PTX plasma PK model, 2) establishing a PTX plasma–PIPN site PK model for each PIPN site, 3) performing a simultaneous analysis of all PIPN sites to derive the PTX plasma-PIPN sites PK model, and 4) building the final PTX PK-NfL model.

#### PTX Plasma PK Model

Linear one-, two-, and three-compartment PK models were evaluated to characterize the total PTX concentration in plasma. Additionally, saturable distribution to the tissues was tested. Considering that the observed nonlinearity in the total PTX concentration–time profiles can be explained to a great extent by its direct proportional binding to the CrEL concentrations due to CrEL micelle entrapment [17], a CrEL PK model was included in the analysis. CrEL concentrations were not measured in rodents. Therefore, the previously established three-compartment CrEL PK model in humans, with saturable elimination and a linear relationship between CrEL and PTX concentrations, was applied [17, 31]. During model building, CrEL model parameters were initially fixed to the previously reported values in humans [17, 31], and then re-estimated one at a time to achieve a better fit for the data observed in rodents. For interspecies extrapolation, allometric scaling by weight was employed for the central and intercompartmental clearance and volume of distribution parameters using exponents of 0.75 and 1, respectively [32, 33].

#### PTX Plasma-PIPN sites PK Model

The total PTX concentration–time profiles in different PIPN sites were described using a PK model, including extracellular and intracellular compartments. The extent of PTX transport across the blood-nervous tissue barriers, i.e., BDB, BNB, and BBB, was determined by the unbound partition coefficient ${K}_{p,uu,x}$, where $x$ represents a specific PIPN site. The ${K}_{p,uu,x}$ was defined as the ratio between the net influx (${CL}_{in,x}$) and efflux clearances (${CL}_{out,x}$) (Eq. 1)

1 $${K}_{p,uu,x}=\frac{{CL}_{in,x}}{{CL}_{out,x}}$$

The PTX transport across the parenchymal cellular barrier was determined by an unbound partition coefficient ${K}_{p,uu,cell,x}$, which is defined as the unbound intracellular-to-extracellular concentration ratio based on the concept developed by Fridén *et al*. [34]. It was calculated assuming rapid equilibrium as follows:

2 $${K}_{p,uu,cell,x}=\frac{{C}_{u,ICF,x}}{{C}_{u,ECF,x}}$$

${C}_{u,ICF,x}$ and ${C}_{u,ECF,x}$ are the intracellular and extracellular (here same as interstitial fluid) unbound PTX concentrations, respectively.

The mass transfer was assumed to be negligible across the nervous tissue barriers. The ${K}_{p,uu,x}$ and ${K}_{p,uu,cell,x}$ were fixed to experimentally determined values [7]. One- and two-compartment models for distribution were evaluated. Finally, the binding of PTX to tubulin within the cell was assessed using an equation for capacity-limited binding (Eq. 3) [35].

3 $${C}_{PTX:tubulin,x}=\frac{{B}_{max,x}\bullet {C}_{u,ICF,x}}{K{d}_{x}+ {C}_{u,ICF,x}}$$

Here, ${C}_{PTX:tubulin,x}$ is the intracellular PTX-tubulin complex concentration, ${B}_{max,x}$ is the maximum tubulin binding capacity in cells, and $K{d}_{x}$ is the dissociation constant for PTX binding to saturable tubulin binding sites in cells.

#### PTX PK-NfL Model Building

Turnover models were evaluated to characterize the NfL concentration–time profiles in serum and CSF after PTX administration. The PTX’s potential impact on the NfL pool in the nervous tissues was assessed on the transition rates i) from the PNS tissues to serum, i.e., central compartment, and ii) from the brain to CSF. Linear and nonlinear relationships (i.e., E_max_ and sigmoidal E_max_ models) were evaluated. Measured median NfL concentrations in saline-control rats in the hippocampus and DRG with SN combined were used to establish the baseline conditions of NfL pools in the CNS and PNS, respectively.

#### Model Development and Statistical Analyses

The nonlinear mixed effect modeling software NONMEM version 7.5.1, executed through Perl-speaks-NONMEM (PsN) version 5.2.6, was used for data analysis and simulations. The first-order conditional estimation method with interaction (FOCEI) was used. R-studio 4.1 was used for data management, model diagnostics, graphical visualization, and evaluations. The selection of the final model was based on; i) OFV (i.e., − 2∙log-likelihood) using the likelihood ratio test to compare between two nested models such that an OFV decrease of 3.84 was considered statistically significant for adding one extra parameter (df = 1, α = 0.05), ii) precision of the parameter estimates, and iii) diagnostic plots. Visual predictive checks (VPCs) were generated by simulating 1,000 datasets to evaluate the models’ predictive performance. One thousand simulation replicates, accounting for parameter uncertainty, were generated by sampling parameter vectors from the posterior distribution acquired by Sampling Importance Resampling (SIR) to assess dose levels of 5, 10, and 15 mg/kg after single and repeated administration in rats, using the same dosing schedule and solution as source 4 (Table I). The PTX PK-NfL model was externally validated using single time-point sNfL data from sources 2, 3 and 4 (Table I).

Statistical analysis of the NfL dataset was performed using GraphPad Prism 9.3.1 for Windows (GraphPad Software, San Diego, CA, United States). Normality was tested with the Shapiro–Wilk test. The statistical significance if differences between NfL concentration in tissues at different time points was tested using mixed-effect analysis.

## Results

### NfL Kinetics in Serum, CSF, and PIPN Sites After Bolus CrEL-PTX

**Fig. 1:**
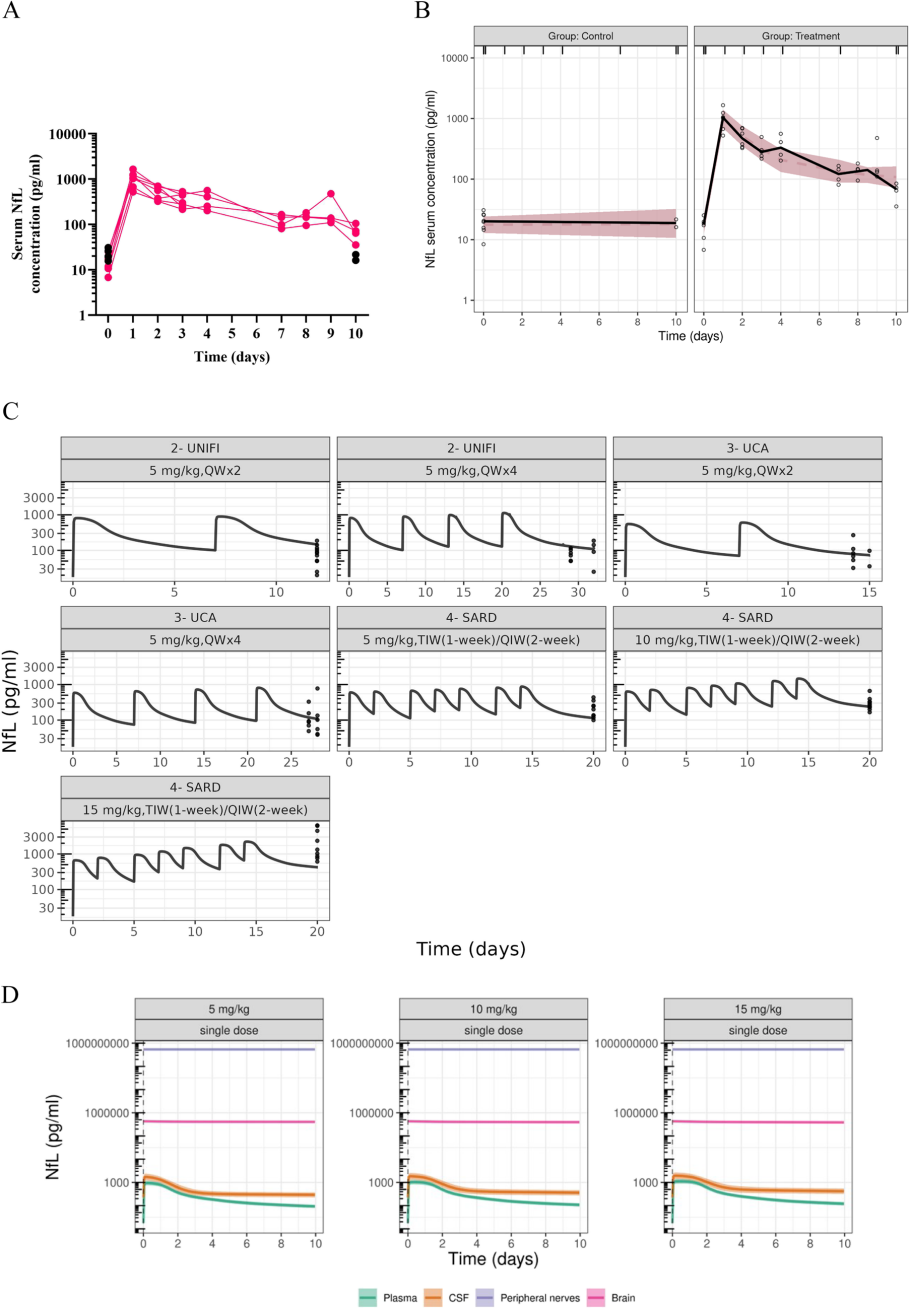
Experimental and model-predicted neurofilament light-chain (NfL) concentration -time profiles in various anatomical sites after paclitaxel (PTX) administration. **(A)** Semilogarithmic plot showing NfL concentrations in serum (pg/mL) over time. Longitudinal daily serum NfL (sNfL) concentrations were measured after administration of a single dose of 10 mg/kg CrEL-PTX (*N* = 7 in total, *N* = 3 terminally sampled at 72 h and *N* = 4 terminally sampled at 240 h) or saline (*N* = 5) bolus injection to the tail vein. **(B)** Visual predictive checks (VPCs) of the NfL kinetics model describing the concentration–time profiles of sNfL in the control and treatment (single 10 mg/kg CrEL-PTX administration) groups. The black dots are the observed concentrations. The solid and dashed lines are the median of the observed and simulated concentrations, respectively. Color-shaded area is the 90% confidence interval of the predicted medians. **(C)** Prediction from the PTX PK- NfL model for the concentration–time profiles of NfL. The solid lines represent the median. The dots are the NfL observations from reported studies, see details in Table I. **(D)** Simulations from the PTX PK- NfL model in rats, with parameter uncertainty, for the total concentration–time profiles of NfL in serum, cerebrospinal fluid (CSF), peripheral nerves, and brain following a single dose of 5, 10, and 15 mg/kg of PTX. The solid lines represent the median, and the shaded areas are the 80% simulation intervals.

To explore the temporal relationship between PTX exposure and NfL release, we assessed NfL in serum, CSF, and PIPN sites concentrations in rodents following a single intravenous dose of 10 mg/kg CrEL-PTX. At the baseline, the average sNfL concentration was 17.6 ± 7.4 pg/mL. The highest sNfL concentration was observed at 24 h post-PTX injection (first sampling time), which was more than 60-fold higher than the baseline. sNfL levels declined gradually but remained elevated at 72 h (335.7 ± 123.9 pg/mL, ~ 19-fold increase) and 240 h (69.3 ± 28.6 pg/mL, ~ fourfold increase) compared to the baseline. By 10 days post-PTX administration, sNfL levels had not returned to baseline (Fig. 1A).

NfL levels in CSF were consistently higher than those in serum. The serum-to-CSF concentration ratio increased from the baseline (0.074 and 0.134, *N* = 2) to 0.267 ± 0.168 at 72 h. A similar tendency was observed in the serum-to-brain ratio. Notably, at 72 h, the mean serum-to-CSF ratio was 668-fold higher than the mean serum-to-brain ratio, suggesting a higher contribution of NfL transport across the blood-CSF barrier. A stable ~ threefold increase in NfL concentrations in CSF post-PTX at 72 and 240 h compared to the baseline has been observed (Table S1).

In nervous tissues, NfL concentration was significantly higher in PNS sites, i.e., SN > DRG >> hippocampus. In PNS tissues, the median NfL concentrations in SN and DRG at baseline were 355.1 µg/mL and 183.6 µg/mL, respectively. There were no significant changes in NfL concentrations in SN and DRG over time. The median NfL concentration in the hippocampus at baseline was 0.43 µg/mL (Table S1). In the hippocampus, a statistically insignificant trend of increase in NfL concentration at 72 and 240 h was observed after treatment compared to the baseline, reaching a median of 0.75 µg/mL (adjusted* p* value = 0.06) and 0.61 µg/mL (adjusted* p* value = 0.37), respectively.

### An Integrated PTX PK-NfL Model Building

**Fig. 2:**
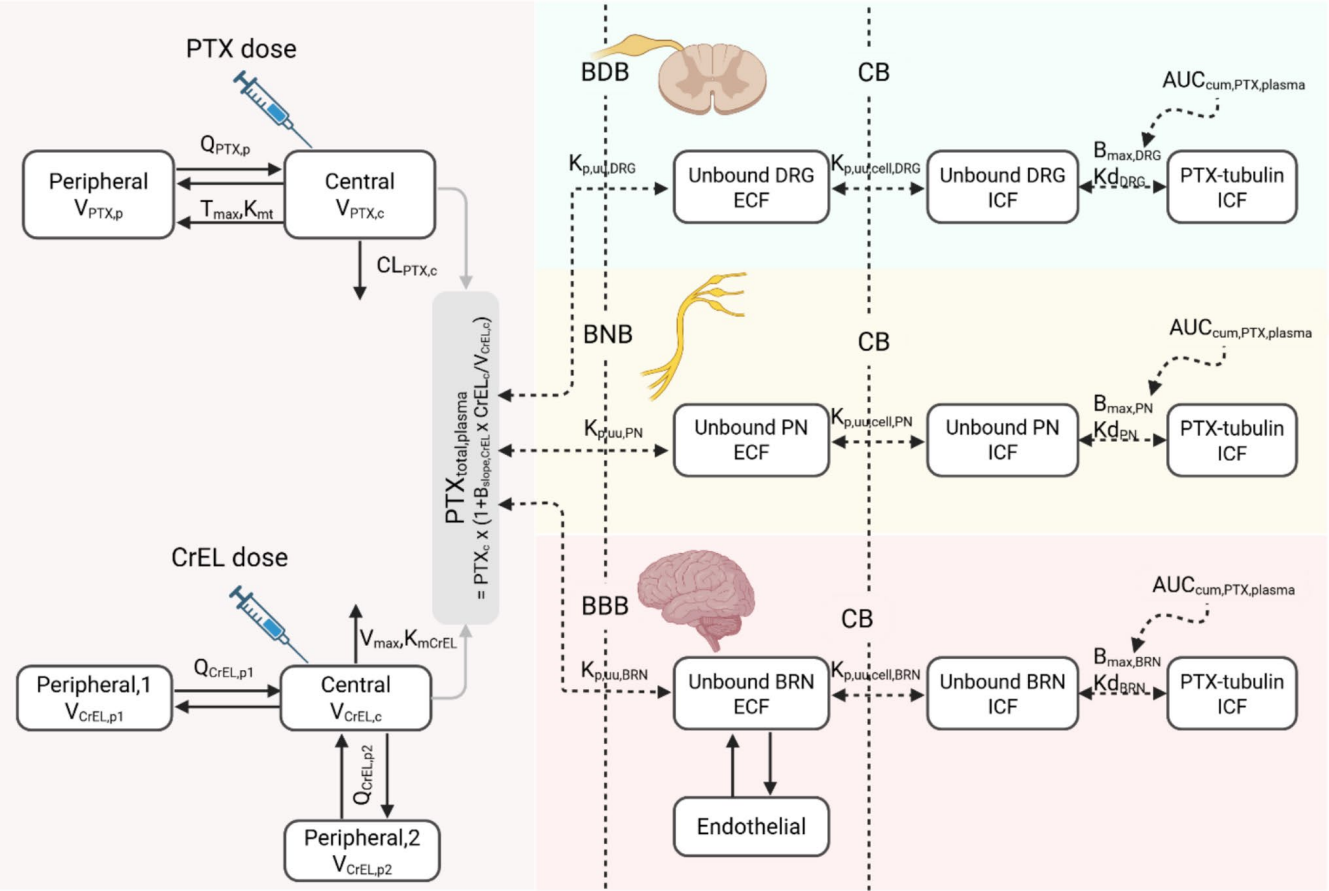
Schematic representation of the PTX plasma–PIPN sites pharmacokinetic (PK) model of paclitaxel and Cremophor EL. Solid black arrows illustrate mass transfer, while dashed black arrows illustrate no mass transfer. The model for plasma (gray) described both PTX and CrEL PK. The PIPN sites PK model characterized the total PTX disposition at different PIPN sites under investigation, namely the dorsal root ganglion (green), peripheral nerves (yellow), and brain (pink). Each nervous tissue model comprised three parts: 1) extracellular unbound PTX, 2) intracellular unbound PTX, and 3) intracellular PTX-tubulin complex. Abbreviations: PIPN – paclitaxel-induced peripheral neurotoxicity, PTX – paclitaxel, CrEL – Cremophor EL, DRG – dorsal root ganglia, PN – peripheral nerves, BRN – brain, ECF – extracellular fluid (here referring to interstitial fluid), ICF – intracellular fluid, BDB – blood-dorsal root ganglion barrier, BNB – blood-nerve barrier, BBB – blood–brain barrier, CB – cellular barrier, Q – intercompartmental clearance, V – volume of distribution, CL – clearance, c – central compartment, p – peripheral compartment, PTXc—the amounts of PTX not unbound to CrEL in central compartment, CrELc – the amount of unbound CrEL in central compartment, T_max_ – maximum transport capacity, K_mt_ – unbound PTX not bound to CrEL concentration at which the transport rate is half-maximal, B_slope,CrEL_ – slope of binding of PTX to CrEL, V_max_ – maximum elimination capacity, K_mCrEL_ – unbound CrEL concentration at which the elimination rate is half-maximal, K_p,uu_ – unbound nervous tissue-to-plasma partition coefficient, K_p,uu,cell_ – unbound ICF-to-ECF concentration ratio, B_max_ – maximum tubulin binding capacity in the cells, K_d_ – dissociation constant for PTX binding to saturable tubulin binding sites in the cells, AUC_cum,PTX,plasma_ – cumulative systemic total PTX plasma exposure. Created in https://BioRender.com.

**Fig. 3:**
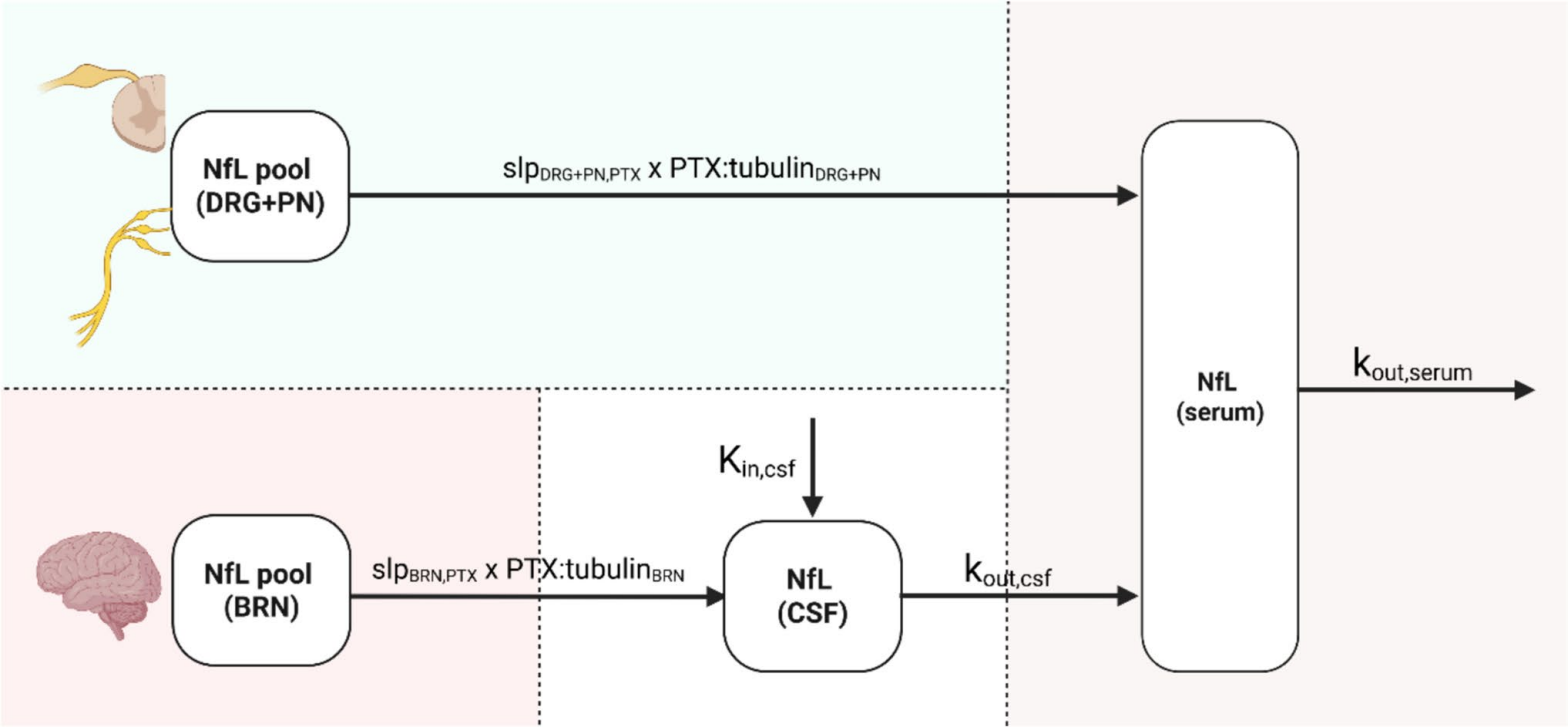
Schematic representation of the neurofilament light chain (NfL) kinetics model. Refer to the model description in the text. Abbreviations: PTX – paclitaxel, DRG – dorsal root ganglia, PN – peripheral nerves, BRN – brain, CSF – cerebrospinal fluid, k_out_ – first order elimination rate constant from respective site, K_in,CSF_ – zero order input rate of NfL into CSF, slp – slope of NfL release under PTX-tubulin complex exposure, PTX:tubulin—intracellular PTX-tubulin complex concentration. Created in https://BioRender.com.

To quantitatively characterize PTX exposure in plasma and anatomically relevant nervous tissues incorporating NfL kinetics after bolus PTX administration, a sequential modeling approach was used. A schematic illustration of the final PTX PK model is presented in Fig. 2 and the NfL kinetics model in Fig. 3. Key model assumptions are listed and evaluated in Table I. The model parameters, along with their estimates and relative standard errors, are summarized in Tables II and III. Model code can be found in supplementary materials.

**Table II:** Key Assumptions Underpinning the PTX PK-NfL Model Building/Use and Their Evaluation

Assumption	Reasoning behind the assumption	Impact of assumption violation (significant, insignificant, unknown)	Probability of assumption violation (likely, unlikely, unknown)
Intracellular PTX-tubulin complex serves as the primary driver of sNfL increase after PTX administration	The PTX–tubulin complex is known to disrupt axonal microtubules and can lead to axonal injury [42]	Significant Given the multifactorial nature of PTX-induced neurotoxicity, additional mechanisms, possibly with different temporal dynamics (e.g., neuroinflammation), may contribute to sNfL elevation	Unknown
Linear relationship between PTX-tubulin complex and NfL kinetics	Within the investigated dose range, model performance was good when a linear relationship was applied	Significant This relationship may be nonlinear, with possible threshold effects or saturation at higher exposure levels. The model may overpredict the NfL release under high-dose or repeated-dose conditions	Unknown
PTX alone drives the increase in NfL leakage	Lack of information on CrEL exposure in PIPN sites and its link to sNfL	Insignificant Although it is suggested that CrEL is neurotoxic [43], no data have been reported on a relationship between CrEL exposure and sNfL	Unlikely
The CrEL vehicle kinetics in rodents resemble kinetics in humans	CrEL PK parameters were extrapolated from the available human model [17] using allometric scaling, due to lack of data in rodents	Unknown While differences in CrEL PK between rodents and humans cannot be entirely ruled out, even after consideration of body weight differences, there is currently no evidence to suggest such discrepancies. Data-driven re-estimation of the maximum elimination capacity of CrEL in rodents was applied	Unknown
PTX does not have an impact on the tissue barriers	No quantitative data on the impact of PTX on the barrier integrity* in vivo *are available	Unknown Preclinical studies suggest that PTX affects endothelial cells and could lead to BBB disruption [44]. The latter, in addition to other neurotoxic mechanisms, may enhance NfL leakage into the circulation The model may attribute changes in NfL leakage solely to axonal injury rather than barrier effects The timing of sNfL increase may be misrepresented, particularly if barrier dysfunction facilitates earlier or greater NfL leakage	Unknown
Slow turnover of NfL with no replacement of NfL in the nervous tissues after PTX administration during the experimental timeframe	Existing literature indicates that NfL is a structural axonal protein with a low basal turnover [45]	Insignificant In cases of severe or widespread axonal damage, intracellular degradation or local clearance of NfL might occur along with compensatory upregulation or downregulation mechanisms in response to PTX administration. A tendency of a non-significant increase in NfL concentrations in the brain was observed in the present study after PTX administration	Unknown

*PTX* paclitaxel, *CrEL* Cremophor, *PK* pharmacokinetics, *NfL* neurofilament light chain

**Table III:** The PTX Plasma – PIPN Sites PK Model’s Parameter Estimates and Their Relative Standard Errors (RSEs)

Parameters	Description	Units	Estimated value^*¶*^	RSE* (%) 95% CI
Plasma PK model				
CL_PTX,c_	clearance of PTX unbound to CrEL	L/hr	1.07	9.7 0.89–1.3
Q_PTX,p_	intercompartmental clearance of PTX unbound to CrEL	L/hr	16.8	12 14–21
V_PTX,c_	central volume of distribution of PTX unbound to CrEL	L	0.559	31 0.25–0.94
V_PTX,p_	peripheral volume of distribution of PTX unbound to CrEL	L	7.49	12 5.9–9.5
T_max_	maximum transport capacity of PTX to the peripheral compartment	mg/hr	0.547	28 0.29–0.93
K_mt_	concentration at which the transport rate is half-maximal	µg/L	0.101	64 0.045–0.29
Q_CrEL,p1_	CrEL intercompartmental clearance 1	L/hr	1.17	FIX [31]
Q_CrEL,p2_	CrEL intercompartmental clearance 2	L/hr	0.479	FIX [31]
V_CrEl,c_	CrEL central volume of distribution	L	4.54	FIX [31]
V_CrEl,p1_	CrEL peripheral volume of distribution 1	L	1.32	FIX [31]
V_CrEl,p2_	CrEL peripheral volume of distribution 2	L	3.53	FIX [31]
V_max_	maximum elimination capacity of CrEL	mL/hr	0.175	11 0.14–0.22
K_mCrEL_	concentration at which the elimination rate is half-maximal	mL/hr	2.57	FIX [31]
B_slope,CrEL_	slope of binding of PTX to the predicted concentrations of CrEL	-	4.46	FIX [17]
RUV_plasma_^**^	residual unexplained variability in total PTX concentrations in plasma	%	36	6.3 0.32–0.41
PIPN site PK model				
f_u_	unbound fraction of PTX in plasma in the absence of CrEL	-	0.069	FIX [7]
f_CrEL_	fraction of PTX bound to CrEL that passes the blood-nervous tissue barriers	-	0.525	17 0.37–0.72
*DRG – Dorsal Root Ganglia*				
K_p,uu,DRG_	unbound DRG-to-Plasma partition coefficient	-	4.25	FIX [7]
K_p,uu,cell,DRG_	unbound ICF-to-ECF concentration ratio in DRG	-	1.45	FIX [7]
CL_out,BDB_	clearance out from DRG	µL/hr	0.577	7.7 0.49–0.66
B_max,DRG,initial_	initial maximum PTX-binding capacity in DRG cells	mg/L^§^	0.287	15 0.21–0.38
Kd_DRG_	dissociation constant for PTX binding to saturable tubulin binding sites in DRG cells	µg/L	0.353	18 0.25–0.5
MAX_DRG_	maximum relative increase in B_max,DRG,initial_	-	14.9	37 7.8–8.6
AUC_50,DRG_	cumulative AUC at which the maximum increase in B_max,DRG,initial_ is half-maximal	mg ∙ hr/L	97.9	27 63–165
h_DRG_	Hill coefficient	-	2.76	20 2–4.4
WT_DRG_	DRG weight	mg	40	FIX [46]
V_ECF,DRG_	ECF volume for DRG	mL/g tissue	0.2	FIX [47, 48]
V_ICF,DRG_	ICF volume for DRG	mL/g tissue	0.8	FIX [47, 48]
RUV_DRG_^**^	residual unexplained variability in total PTX concentrations in DRG	%	49	7.9 (44–59)
*PN –Peripheral Nerves*				
K_p,uu,PN_	unbound PN-to-Plasma partition coefficient	-	4.48	FIX [7]
K_p,uu,cell,PN_	unbound ICF-to-ECF concentration ratio in PN	-	0.55	FIX [7]
CL_out,BNB_	clearance out from PN	µL/hr	772.5	64 387–2074
B_max,PN,initial_	initial maximum PTX-binding capacity in PN cells	mg/L^§^	0.144	18 0.1–0.2
Kd_PN_	dissociation constant for PTX binding to saturable tubulin binding sites in PN cells	µg/L	0.353	18 0.25–0.5
SLP_PN_	coefficient of the exponential increase of B_max,PN,initial_ as a function of cumulative AUC	L/mg/hr	0.0194	7.4 0.017–0.022
WT_PN_	PN weight	mg	2781	FIX^§^
V_ECF,PN_	ECF volume for PN	mL/g tissue	0.5	FIX^#^
V_ICF,PN_	ICF volume for PN	mL/g tissue	0.5	FIX^#^
RUV_PN_^**^	residual unexplained variability in total PTX concentrations in PN	%	69	8.7 (60–83)
*BRN—brain*				
K_p,uu,BRN_	unbound brain-to-plasma partition coefficient	-	0.032	FIX [7]
K_p,uu,cell,BRN_	unbound ICF-to-ECF concentration ratio in brain	-	11.2	FIX [7]
CL_out,BBB_	clearance out from brain	µL/hr	23.7	22 15–34
Q_end,BRN_	intercompartmental clearance of PTX in ECF	µL/hr	6.47	30 4–12
V_end,BRN_	volume of distribution of the endothelial compartment	mL	3.23	125 1–15
B_max,BRN,initial_	initial maximum PTX-binding capacity in brain cells	mg/L^§^	0.0259	21 0.017–0.039
Kd_BRN_	dissociation constant for PTX binding to saturable tubulin binding sites in brain cells	µg/L	4.208	FIX [35]
MAX_BRN_	maximum relative increase in B_max,BRN,initial_	-	14.9	37 7.8–8.6
AUC_50,BRN_	cumulative AUC at which the maximum increase in B_max,BRN,initial_ is half-maximal	mg ∙ hr/L	97.9	27 63–165
h_BRN_	Hill coefficient	-	2.76	20 2–4.4
WT_BRN_	brain weight	g	0.4 (mice) 1.8 (rat)	FIX (46)
V_ECF,BRN_	ECF volume for BRN	mL/g tissue	0.2	FIX [47, 48]
V_ICF,BRN_	ICF volume for BRN	mL/g tissue	0.8	FIX [47, 48]
RUV_BRN_^**^	residual unexplained variability in total PTX concentrations in the brain	%	42	9.8 (36–52)

*PTX*-paclitaxel, *PIPN* paclitaxel-induced peripheral neurotoxicity, *PK* pharmacokinetics

*obtained from Sampling Importance Resampling (SIR). **additive residual error on log-transformed data. ^*§*^determined based on that 0.93% of the body weight in rats, similar to the estimate in humans [49]. ^*#*^determined according to data published at https://www.nysora.com/topics/anatomy/connective-tissues-peripheral-nerves/ describing that up to 50% of the cross-sectional area is made up of non-neural tissue, including endoneural fluid and connective stroma and it can vary between different nerves. ^¶^clearance and volume of distribution parameters were estimated at an average rat body weight of 0.3 kg.^*§*^ The tissue density is assumed to be 1 g/mL

**Table IV:** The NfL Kinetics Model’s Parameter Estimates and Their Relative Standard Errors (RSEs)

Parameters	Description	Units	Estimated value	RSE* (%) (95% CI)
NfL_baseline,serum_	baseline NfL concentration in serum	pg/mL	17.64	12 (14—22)
NfL_baseline,csf_	baseline NfL concentration in CSF	pg/mL	222.5	20 (147—319)
NfL_baseline,brain_	baseline NfL concentration in the brain	pg/mL	0.43 × 10^6^	FIX
NfL_baseline,PN_	baseline NfL concentration in peripheral tissues	pg/mL	538.74 × 10^6^	FIX
slp_NfL,brain,PTX_	slope of NfL release into CSF under PTX-tubulin complex exposure in the brain	-	0.029 × 10^9^	9 (0.025—0.034)
k_out,serum_	first-order elimination rate constant of NfL from serum	1/day	10.1	FIX [27]^§^
V_serum_	serum volume	mL	5	FIX [50]
V_csf_	CSF volume	mL	0.25	FIX [51]
RUV_serum_***	residual unexplained variability in NfL concentrations in serum	%	46	10 (39—58)
RUV_csf_***	residual unexplained variability in NfL concentrations in CSF	%	37	47 (24—90)

*NfL* neurofilament light chain, *CSF* cerebrospinal fluid, *PTX* paclitaxel, *DRG* dorsal root ganglia, *PN* peripheral nerves

*obtained from Sampling Importance Resampling (SIR). **slope of NfL release into serum under PTX-tubulin complex exposure in DRG and PN, slp_Nfl,DRG+PN,PTX_, was assumed to be the same as slp_NfL,brain,PTX_. *** additive residual error on log-transformed data. ^*§*^allometric scaling with body weight was applied with a fixed exponent, −0.25, using 70 (kg) as a standard reference body weight for an adult human

### PTX Plasma PK Model

The developed PK model successfully described the total plasma PTX concentration–time profiles in rats and mice after administration of various PTX doses (Fig. S1A). The data was best described by a two-compartment model with a mixture of unsaturable and saturable distribution to the peripheral compartment (Eq. 4), where ${T}_{max}$ is the maximum transport capacity and ${K}_{mt}$ is the not bound to CrEL PTX concentration ($\frac{PT{X}_{c}}{{V}_{PTX,c}}$) at which the transport rate is half-maximal.

4 $${Q}_{non-linear ,PTX}=\frac{{T}_{max}}{{K}_{mt}+ \frac{PT{X}_{c}}{{V}_{PTX,c}}}$$

where $Q$ is the inter-compartment clearance.

For the CrEL model, re-estimation of the maximum elimination capacity of CrEL (${V}_{max}$) from the whole observed rodent dataset resulted in a statistically significant improvement in the model fit with an estimated value of 0.175 mL/hr.

### PTX Plasma-PIPN Sites PK Model

The total PTX concentration at each PIPN site was the sum of the amounts of the extracellular and intracellular unbound PTX, and the intracellular PTX-tubulin complex, divided by the PIPN site’s physiological volume. The final model captured nonlinear plasma kinetics, tissue-specific PTX distribution, and dynamic changes in tubulin binding capacity, supporting its utility for downstream PKPD modeling of PIPN-related biomarker changes.

The unbound PTX amount in the extracellular fluid (ECF) originates from the passage of unbound PTX and a fraction of PTX bound to CrEL across the barriers (Fig. 2). The PTX-CrEL binding model is presented in Eq. 5. The unbound PTX transport across the blood-nervous tissue barriers was characterized, with $C{L}_{out,x}$ (Eq. 6) estimated to 0.577, 773, and 23.7 µL/hr for DRG, PN, and brain, respectively, in a 0.3 kg rat. Allometric scaling was applied to estimate clearance and volume of distribution parameters in mice.

5 $$PT{X}_{PTX:CrEL,c}=PT{X}_{c}\bullet {B}_{slope,CrEL} \bullet \frac{{CrEL}_{c}}{{V}_{CrEL,c}}$$

6 $$\frac{d \left(PT{X}_{x}\right)}{dt}=\left( PT{X}_{c}\bullet {f}_{u}+ PT{X}_{PTX:CrEL,c}\bullet {f}_{CrEL}\right)\bullet \frac{C{L}_{in,x}}{{V}_{PTX,c}}- PT{X}_{x}\bullet \frac{C{L}_{out,x}}{{V}_{ECF,x}}$$

where $PT{X}_{c}$ and $PT{X}_{PTX:CrEL,c}$ are the amounts of PTX not bound and bound to CrEL in the central compartment (i.e., plasma), respectively, B_slope,CrEL_ is the slope for binding of PTX to CrEL, CrEL_c_ is the amount of CrEL not bound to PTX and V_CrEL,c_ is CrEL the volume of distribution of the central compartment. $PT{X}_{x}$ is the amount of unbound PTX in ECF of nervous tissue $x$, $PT{X}_{c}$ ${f}_{u}$ is the experimentally determined unbound fraction of PTX in plasma in the absence of CrEL, ${f}_{CrEL}$ is the fraction of PTX bound to CrEL that crosses the blood-nervous tissue barrier, ${V}_{PTX,c}$ is the central volume of distribution of PTX unbound to CrEL and ${V}_{ECF}$ is the ECF volume. For brain tissue, a two-compartment model best described the concentration–time profiles observed *in vivo*.

When total intracellular PTX concentration in PIPN sites was examined, a discrepancy was observed between single and repeated dosing regimens likely governed by specific binding of PTX to tubulin. Repeated systemic exposure to PTX had a statistically significant influence on maximum tubulin binding capacity ${B}_{max,x}$. This was described as an increase from the initial maximum binding capacity (${B}_{max,x,initial}$) in DRG and brain tissues according to a Hill function, and in PN using an exponential function, derived by the cumulative systemic total PTX plasma exposure, i.e., ${AUC}_{cum,PTX,plasma}$ (Eqs. 7–8).

7 $${B}_{max,x}= {B}_{max,x,initial}\bullet (1+\frac{{{{MAX}_{x}\bullet AUC}_{cum,PTX,plasma}}^{{h}_{x}}}{{{AUC50}_{x}}^{{h}_{x}}+ {AU{C}_{cum,PTX,plasma}}^{{h}_{x}}} )$$

8 $${B}_{max,PN}= {B}_{max,PN,initial}\bullet {e}^{({SLP}_{PN}\bullet AU{C}_{cum,PTX,plasma})}$$

where ${MAX}_{x}$ represents the maximum increase from ${B}_{max,x,initial}$, ${AUC50}_{x}$ represents the $AU{C}_{cum,PTX,plasma}$ at which the maximum increase in ${B}_{max,x,initial}$ is half-maximal, ${h}_{x}$ represents the Hill coefficient, and ${SLP}_{PN}$ is the coefficient of the exponential increase of ${B}_{max,x,initial}$ as a function of $AU{C}_{cum,PTX,plasma}$. Although extrapolation beyond evaluated doses requires caution, the model is stable across biologically relevant exposure ranges and fit for its intended use.

**Fig. 4:**
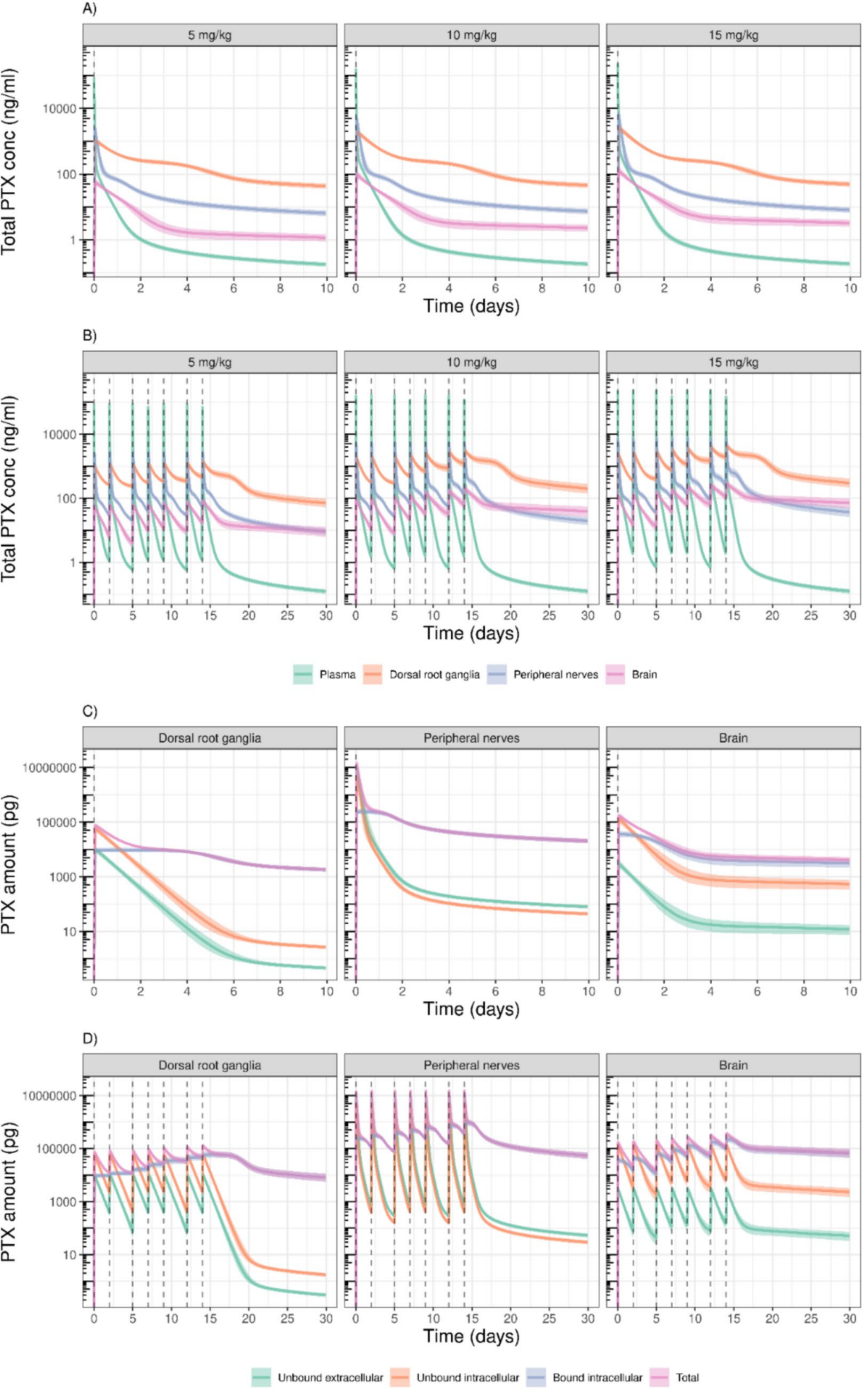
Simulations with parameter uncertainty from the PTX plasma–PIPN sites pharmacokinetic (PK) model in rats. The total concentration–time profiles of paclitaxel (PTX) in plasma, dorsal root ganglia, peripheral nerves, and brain after **(A)** single dose and **(B)** multiple dose (three times a week at week 1 and four times a week at week 2) administration of 5, 10, and 15 mg/kg. The amount-time profiles of PTX in the dorsal root ganglia, peripheral nerves, and brain following **(C)** a single dose and **(D)** multiple doses of the same regimen of 10 mg/kg. The solid lines represent the median, and the shaded areas are the 80% simulation intervals. The vertical dashed lines are the PTX dosing times

Using the final PTX PK model, simulations were performed to explore PTX distribution dynamics across dosing regimens and tissue compartments. Total PTX concentration–time profiles following single and multiple intravenous administrations (5, 10, and 15 mg/kg; three times in week 1 and four times in week 2) illustrated rapid plasma decline and marked accumulation in PIPN sites (Fig. 4A, B). Among these, the DRG exhibited the highest concentrations, followed by PN, except after multiple dosing, where brain concentrations surpassed those in PN approximately five days after the last dose. To account for differences in tissue mass, simulations of PTX amounts (rather than concentrations) were conducted across intracellular and extracellular compartments following 10 mg/kg PTX (Fig. 4C, D). The simulation-based diagnostics for the total PTX concentration–time profiles in PIPN sites showed a good model predictive performance, with observations corresponding well with the 90% of the confidence intervals of the predicted medians (Fig. S1B-S1D). The majority of PTX is localized intracellularly, with the largest overall amounts observed in peripheral nerves, followed by the brain and DRG. Intracellularly bound PTX, representing PTX–tubulin complex and serving as the driver of NfL release in our model, was most abundant in nerves after single dosing (Fig. S2). However, following repeated administration, brain-bound PTX eventually exceeded nerve levels, consistent with slow elimination of PTX from the brain, i.e., $C{L}_{out}$ of 773 *vs* 23.7 µL/hr for PN and brain, respectively. These simulations highlighted the tissue-specific PK differences.

### PTX PK-NfL Model

To quantify the link between PTX exposure and NfL biomarker kinetics, we successfully developed a PTX PK-NfL model (Fig. 3). The model incorporated two anatomical NfL pools, brain and peripheral nervous tissues, i.e., DRG and PN combined (Eqs. 9–10). Two turnover models were used to characterize NfL kinetics in the CSF and serum (Eqs. 11–12).

9 $$\frac{d \left(Nf{L}_{brain}\right)}{dt}=- {slp}_{brain,PTX}\bullet PT{X:tubulin}_{BRN}$$

10 $$\frac{d \left(Nf{L}_{DRG+PN}\right)}{dt}=- {slp}_{DRG+PN,PTX}\bullet PT{X:tubulin}_{DRG+PN}$$

11 $$\frac{d \left(Nf{L}_{csf}\right)}{dt}={K}_{in,csf}+{slp}_{brain,PTX}\bullet PT{X:tubulin}_{BRN}- {k}_{out,csf}\bullet Nf{L}_{csf}$$

12 $$\frac{d \left(Nf{L}_{serum}\right)}{dt}={k}_{out,csf}\bullet Nf{L}_{csf}+{slp}_{DRG+PN,PTX}\bullet PT{X:tubulin}_{DRG+PN}- {k}_{out,serum}\bullet Nf{L}_{serum}$$

where $Nf{L}_{csf}$ and $Nf{L}_{serum}$ represent the NfL amounts in CSF and serum, respectively, ${K}_{in,csf}$ is the zero order input rate of NfL into CSF, ${k}_{out,csf}$ is the first order transfer rate constant of NfL from CSF to serum, ${k}_{out,serum}$ is the first order elimination rate of NfL from serum, and ${slp}_{brain,PTX}$ and ${slp}_{DRG+PN,PTX}$ are the slopes of NfL input from brain to CSF, and DRG and PN to serum, respectively, under PTX-tubulin complex exposure in brain ($PT{X:tubulin}_{BRN}$), and DRG and PN ($PT{X:tubulin}_{DRG+PN}$), respectively.

The PTX-induced increase in NfL leakage, from the brain to CSF and from the DRG and PN to serum, was best described by a linear relationship. Under baseline conditions without PTX exposure, steady-state kinetics was assumed (Eqs. 13–14).

13 $${K}_{in,csf}= {NfL}_{baseline,csf}\bullet {k}_{out,csf}$$

14 $${k}_{out,csf}={k}_{out,serum}\bullet \left(\frac{{NfL}_{baseline,serum}}{{NfL}_{baseline,csf}}\right)$$

The VPCs for the NfL concentration–time profiles (Fig. 1B, Fig. S3) showed good agreement with the observed data. Additionally, the final model demonstrated an acceptable predictive performance by capturing the observations in the external dataset, while underpredicting the NfL concentrations after high, 15 mg/kg dose (Fig. 1C). Simulations of the temporal dynamics of NfL release, following single intravenous doses of 5, 10, and 15 mg/kg PTX (Fig. 1D), illustrated that NfL concentrations in PN and brain were substantially higher than those in serum and CSF, and remained nearly constant throughout the 10-day simulation period. For the first three days post-dose, serum and CSF profiles were parallel, after which serum NfL declined more rapidly than CSF NfL. In line with experimental observations, neither serum nor CSF NfL returned to baseline within the 10-day post-dose window. Simulations also suggested that NfL leakage from PN could be the main contributor to the elevated serum levels after single dosing (Fig. S4).

## Discussion

We successfully developed an integrated PTX PK-NfL model that captures PTX and NfL kinetics in plasma and PIPN-relevant sites, such as DRG, SN, and brain, in rodents. This provided new insights into PTX distribution and its temporal behavior after different doses and dosing regimens. By connecting PTX-tubulin complex formation in PIPN sites, this model accurately predicted sNfL kinetics observed *in vivo*. This was possible due to the de novo generated longitudinal NfL data after PTX administration. While the model underpredicted sNfL levels after chronic PTX dosing based on the available dataset, it offered a valuable foundation for characterizing the PTX–sNfL relationship and guiding future context-of-use biomarker-informed approaches to PIPN.

This is the first study examining NfL concentrations in PIPN sites after PTX administration. We quantified NfL in the DRG, SN, and hippocampus at baseline and at 72 and 240 h after CrEL–PTX dosing and observed no significant changes over time. Notably, hippocampal NfL concentrations in control rats were ca tenfold lower than reported values in healthy humans [36]. For modeling purpose, when quantifying the NfL pool in the CNS, we have assumed a similar NfL distribution across brain regions, although previous findings indicated slight inter-region variability in the human brain [36]. Currently, data on the abundance of NfL in rodent PNS tissues are limited to Western blot analysis [37, 38], with no absolute values reported. In this study, we measured NfL levels in DRG and peripheral nerves, which were 460 and 887-fold higher than in the brain, respectively. Our data suggest that the NfL pool in the PNS is substantially greater than that in the brain of rodents. When evaluating individual contributions of PIPN sites to sNfL kinetics, the peripheral nerves were found to be the most significant contributor in all investigated PTX doses (Fig. S4). Interestingly, the model simulations suggested a higher relative contribution of the brain to the observed sNfL kinetics compared with the DRG, which may be partially attributable to differences in tissue size. The brain contribution to sNfL was further emphasized by the observed ~ threefold increase in CSF NfL at 72 and 240 h after PTX administration compared to baseline. The NfL leakage from the brain to serum may occur across the blood-CSF barrier and/or the BBB. Unraveling the relationship between NfL concentrations in brain interstitial fluid and CSF is key to investigating the role of brain barriers in CSF NfL kinetics. Further studies are needed to mechanistically understand the cause of CSF NfL increase, which might be due to axonal damage and/or changes in brain barrier function after PTX administration.

Along with NfL examination at PIPN sites, the evaluation of sNfL kinetics after a single 10 mg/kg PTX dose was performed. A prolonged NfL leakage into serum was observed, with levels remaining elevated for at least 10 days. This delayed return of sNfL to baseline mirrors trends observed clinically after PTX discontinuation [22], suggesting that PTX-induced axonal injury may have long-lasting biomarker signatures. Previously, sNfL increase compared to the baseline was observed after repeated dosing regimens of PTX using sparse sampling [11, 15]. Using a longitudinal sampling approach, we have expanded the knowledge of sNfL kinetics after bolus PTX administration, therefore increasing the temporal resolution of the sNfL kinetics. Interestingly, we have observed maximal sNfL concentration at 24 h, the first sampling time, indicating rapid changes. Due to this unexpected observation, it is highly recommended to include sNfL measurements at earlier time points after PTX administration. To mechanistically interpret the sNfL kinetics and establish a quantitative PKPD relationship, it was essential to characterize PTX exposure at systemic and tissue levels. To our knowledge, no previous PK model has explicitly linked plasma concentrations of PTX to drug exposure at the sites implicated in PIPN. Unlike models focusing solely on systemic exposure, our approach integrates compartments representing the neurotoxic target sites. This enables quantitative prediction of local drug kinetics, providing a mechanistic basis for exploring exposure–toxicity relationships and optimizing dosing strategies to reduce PIPN risk. To test this approach, we are currently expanding the PTX PK-NfL model by performing interspecies scaling to humans and validating the predictions with clinical systemic PK and sNfL data, as well as relating those variables to PIPN severity measurements.

The plasma PK model captures two nonlinear behaviors of PTX: one related to micelle trapping by CrEL [39], and the other attributed to distribution. The latter is presumably mediated by a combination of active influx (e.g., via OATP1B1 and OATP1B3: solute carrier organic anion-transporting polypeptide B1 and B3) and efflux (e.g., via P-glycoprotein) transport, previously reported for PTX [40, 41]. Given the unavailability of CrEL (i.e., the PTX formulation vehicle) concentrations in rodents, its model structure was adapted from a published human model [31] with parameters allometrically scaled. Yet, the maximum elimination capacity of CrEL was re-estimated, reflecting potential interspecies differences in CrEL elimination capacity of 0.175 mL/hr in rats *vs* 0.64 mL/hr in humans [31]. The distribution of PTX to PIPN sites is a complex process, governed by, but not limited to, passive, active influx/efflux transport [7]. The available preclinical data from multiple studies with sparse sampling enabled us to explore PTX distribution to PIPN sites. Additionally, the transport between different anatomical locations was guided by previously generated experimental data on the extent of unbound PTX distribution across BDB, BNB, and BBB as well as cellular barrier transport in DRG, SN, and brain [7]. Together, these data allowed us to characterize tissue-specific distribution profiles in PIPN sites following various doses and dosing regimens using modeling approaches. The PTX plasma–PIPN–sites PK model successfully described the prolonged PTX accumulation in the PNS tissues as reported among others by Wozniak *et al*. [5]. In their study, following a single intravenous dose, the drug was rapidly cleared from plasma but persisted in the DRG and SN for more than 72 h. After a 2-week dosing regimen, the drug remained in peripheral nerves for weeks, despite rapid plasma clearance, with functional measures showing severe neurotoxicity [5]. The persistence of PTX in PIPN sites results from an interplay between active transport across biological membranes [40, 41], specific (e.g., tubulin) [19] and non-specific binding. The binding of PTX to tubulin within cells was implemented using a capacity-limited binding equation, which successfully captured the increase in the maximum binding capacity of PTX to tubulin following repeated systemic exposure. This finding aligns with the study by Kuh *et al*. [35], who, using a data-driven computational model of PTX intracellular PK, reported a time- and concentration-dependent increase in tubulin levels.

The developed PTX PK-NfL model successfully quantified the increases in CSF and serum NfL levels, attributed to leakage from the brain and PNS tissues induced by PTX–tubulin complex formation. A crucial assumption of this analysis is that PTX–tubulin complex formation drives sNfL kinetics. The latter is likely to occur through microtubule stabilization [1, 21], a mechanism linked to axonal degeneration *in vitro* [42]. The toxic effect of tubulin-bound PTX has been previously described [1], further strengthening the validity of this assumption. The experimental timeframe did not permit precise estimation of NfL turnover parameters, because NfL turnover in CSF [28] and plasma [27] is substantially faster than that of the clearance of the PTX–tubulin complex within nervous tissues, shown to be persistent for more than 10 days (Fig. S2). These values were therefore fixed to literature values (Table 3). In addition, the model assumed an instantaneous NfL release upon PTX exposure, as no data were collected within a 0–24 h post-dose window. Due to a lack of supporting data, assumptions could not be fully validated. Although histopathological evaluations and behavioral assays were explored, the developed PTX PK–NfL model ultimately did not incorporate PIPN assessment due to the scarcity of data [11, 12]. The availability of longitudinal PD observations following PTX treatment in rodents would be critical to further strengthening the model’s ability to link PTX PK with NfL kinetics and PIPN development.

## Conclusions

In this work, we present the first PTX PK-NfL model that mechanistically links PTX accumulation in PIPN sites such as DRG, PN, and the brain to its effect on NfL kinetics. The model quantifies the impact of the PTX–tubulin complex on NfL leakage from PIPN sites, and explores the contributions of CNS and PNS NfL pools to sNfL kinetics. The model is capable of predicting not only plasma exposure, but also extracellular and intracellular PTX concentrations, including tubulin binding over time. Our model-based simulations provided new evidence on PTX accumulation after different dosing regimens of CrEL-PTX in PIPN sites. Despite the different extents of distribution to the PIPN sites and distinct systemic PK parameters for various PTX formulations [8, 18], the developed model can be adapted to accommodate PTX formulation-specific characteristics. Generated knowledge could help to establish NfL as a biomarker for PTX-induced axonal damage and apply the developed PTX PK-NfL model for translation of *in vivo* preclinical data on NfL kinetics to clinical applications.

## Supplementary Information

Below is the link to the electronic supplementary material.

ESM 1 (PDF 124 KB)

ESM 2 (PDF 1.00 MB)

## Data Availability

The datasets used and/or analysed during the current study are available from the corresponding author on reasonable request.

## References

[1] Chen X, Gan Y, Au NPB, Ma CHE. Current understanding of the molecular mechanisms of chemotherapy-induced peripheral neuropathy. Front Mol Neurosci. 2024;17: 1345811. 38660386 10.3389/fnmol.2024.1345811 PMC11039947

[2] Seretny M, Currie GL, Sena ES, Ramnarine S, Grant R, MacLeod MR, *et al*. Incidence, prevalence, and predictors of chemotherapy-induced peripheral neuropathy: a systematic review and meta-analysis. Pain. 2014;155(12):2461–70. 25261162 10.1016/j.pain.2014.09.020

[3] Jordan B, Margulies A, Cardoso F, Cavaletti G, Haugnes HS, Jahn P, *et al*. Systemic anticancer therapy-induced peripheral and central neurotoxicity: ESMO-EONS-EANO clinical practice guidelines for diagnosis, prevention, treatment and follow-up. Ann Oncol. 2020;31(10):1306–19. 32739407 10.1016/j.annonc.2020.07.003

[4] Omran M, Belcher EK, Mohile NA, Kesler SR, Janelsins MC, Hohmann AG, *et al*. Review of the role of the brain in chemotherapy-induced peripheral neuropathy. Front Mol Biosci. 2021;8: 693133. 34179101 10.3389/fmolb.2021.693133 PMC8226121

[5] Wozniak KM, Vornov JJ, Wu Y, Nomoto K, Littlefield BA, DesJardins C, *et al*. Sustained accumulation of microtubule-binding chemotherapy drugs in the peripheral nervous system: correlations with time course and neurotoxic severity. Cancer Res. 2016;76(11):3332–9. 27197173 10.1158/0008-5472.CAN-15-2525 PMC4891279

[6] Nakamura I, Ichimura E, Goda R, Hayashi H, Mashiba H, Nagai D, *et al*. An in vivo mechanism for the reduced peripheral neurotoxicity of NK105: a paclitaxel-incorporating polymeric micellar nanoparticle formulation. Int J Nanomed. 2017;12:1293–304. 10.2147/IJN.S114356 PMC5317268 28243090

[7] Hu Y, Girdenyté M, Roest L, Liukkonen I, Siskou M, Bällgren F, *et al*. Analysis of the contributing role of drug transport across biological barriers in the development and treatment of chemotherapy-induced peripheral neuropathy. Fluids Barriers CNS. 2024;21(1): 13. 38331886 10.1186/s12987-024-00519-7 PMC10854123

[8] Girdenytė M, Hu Y, Ginosyan A, Hammarlund-Udenaes M, Loryan I. Formulation-dependent differences in paclitaxel distribution to anatomical sites relevant to chemotherapy-induced peripheral neuropathy. Front Pharmacol. 2024;15: 1486686. 39568585 10.3389/fphar.2024.1486686 PMC11576287

[9] Li F, Zhang H, He M, Liao J, Chen N, Li Y, *et al*. Different nanoformulations alter the tissue distribution of paclitaxel, which aligns with reported distinct efficacy and safety profiles. Mol Pharm. 2018;15(10):4505–16. 30180593 10.1021/acs.molpharmaceut.8b00527 PMC8851508

[10] Sparreboom A, Scripture CD, Trieu V, Williams PJ, De T, Yang A, et al. Comparative Preclinical and Clinical Pharmacokinetics of a Cremophor-Free, Nanoparticle Albumin-Bound Paclitaxel (ABI-007) and Paclitaxel Formulated in Cremophor (Taxol). Clin Cancer Res. 2005;11(11):4136–43. 15930349 10.1158/1078-0432.CCR-04-2291

[11] Balayssac D, Busserolles J, Broto C, Dalbos C, Prival L, Lamoine S *et al.* Neurofilament light chain in plasma as a sensitive diagnostic biomarker of peripheral neurotoxicity: In Vivo mouse studies with oxaliplatin and paclitaxel-NeuroDeRisk project. Biomedicine & Pharmacotherapy. 2023;167:115535. 10.1016/j.biopha.2023.115535 37738793

[12] Micheli L, Balayssac D, Busserolles J, Dalbos C, Prival L, Richard D, Quintana M, Di Cesare Mannelli L, Toti A, Ciampi C, Ghelardini C, Vlasakova K, Glaab WE, Hu Y, Loryan I, Perrault O, Slaoui M, Wuersch K, Johnson E, Frieauff W, Penraat K, Brees D, Dubost V, Theil D The challenge to identify sensitive safety biomarkers of peripheral neurotoxicity in the rat: a collaborative effort across industry and academia (IMI NeuroDeRisk project). Toxicology. 2024:153998. 10.1016/j.tox.2024.153998 10.1016/j.tox.2024.153998 39551123

[13] Balayssac D, Durif J, Lambert C, Dalbos C, Chapuy E, Etienne M, *et al*. Exploring serum biomarkers for neuropathic pain in rat models of chemotherapy-induced peripheral neuropathy: a comparative pilot study with oxaliplatin, paclitaxel, bortezomib, and vincristine. Toxics. 2023. 10.3390/toxics11121004. 38133405 10.3390/toxics11121004 PMC10747971

[14] Peters CM, Jimenez-Andrade JM, Jonas BM, Sevcik MA, Koewler NJ, Ghilardi JR, *et al*. Intravenous paclitaxel administration in the rat induces a peripheral sensory neuropathy characterized by macrophage infiltration and injury to sensory neurons and their supporting cells. Exp Neurol. 2007;203(1):42–54. 17005179 10.1016/j.expneurol.2006.07.022

[15] Meregalli C, Fumagalli G, Alberti P, Canta A, Chiorazzi A, Monza L,* et al*. Neurofilament light chain: a specific serum biomarker of axonal damage severity in rat models of chemotherapy-induced peripheral neurotoxicity. Arch Toxicol. 2020;94(7):2517–22. 32333051 10.1007/s00204-020-02755-w

[16] Henningsson A, Karlsson MO, Viganò L, Gianni L, Verweij J, Sparreboom A. Mechanism-based pharmacokinetic model for paclitaxel. J Clin Oncol. 2001;19(20):4065–73. 11600609 10.1200/JCO.2001.19.20.4065

[17] Henningsson A, Sparreboom A, Sandström M, Freijs A, Larsson R, Bergh J, *et al*. Population pharmacokinetic modelling of unbound and total plasma concentrations of paclitaxel in cancer patients. Eur J Cancer. 2003;39(8):1105–14. 12736110 10.1016/s0959-8049(03)00126-6

[18] Chen N, Li Y, Ye Y, Palmisano M, Chopra R, Zhou S. Pharmacokinetics and pharmacodynamics of nab-paclitaxel in patients with solid tumors: disposition kinetics and pharmacology distinct from solvent-based paclitaxel. J Clin Pharmacol. 2014;54(10):1097–107. 24719309 10.1002/jcph.304 PMC4302229

[19] Karlsson MO, Molnar V, Freijs A, Nygren P, Bergh J, Larsson R. Pharmacokinetic models for the saturable distribution of paclitaxel. Eur J Cancer. 1999;27(10):1220–3. 10497151

[20] Rodwin RL, Siddiq NZ, Ehrlich BE, Lustberg MB. Biomarkers of chemotherapy-induced peripheral neuropathy: current status and future directions. Front Pain Res. 2022;3:864910. 10.3389/fpain.2022.864910 PMC8963873 35360655

[21] Huehnchen P, Schinke C, Bangemann N, Dordevic AD, Kern J, Maierhof SK, *et al*. Neurofilament proteins as a potential biomarker in chemotherapy-induced polyneuropathy. JCI insight. 2022;7(6). 10.1172/jci.insight.154395 PMC8986065 35133982

[22] Mortensen C, Steffensen KD, Simonsen E, Herskind K, Madsen JS, Olsen DA, *et al*. Neurofilament light chain as a biomarker of axonal damage in sensory neurons and paclitaxel-induced peripheral neuropathy in patients with ovarian cancer. Pain. 2023;164(7):1502–11. 10.1097/j.pain.0000000000002840 10.1097/j.pain.0000000000002840 36508173

[23] Karteri S, Bruna J, Argyriou AA, Mariotto S, Velasco R, Alemany M, *et al*. Prospectively assessing serum neurofilament light chain levels as a biomarker of paclitaxel-induced peripheral neurotoxicity in breast cancer patients. J Peripher Nerv Syst. 2022;27(2):166–74. 35384143 10.1111/jns.12493

[24] Alberti P, Semperboni S, Cavaletti G, Scuteri A. Neurons: the interplay between cytoskeleton, ion channels/transporters and mitochondria. Cells. 2022. 10.3390/cells11162499. 36010576 10.3390/cells11162499 PMC9406945

[25] Khalil M, Teunissen CE, Otto M, Piehl F, Sormani MP, Gattringer T, *et al*. Neurofilaments as biomarkers in neurological disorders. Nat Rev Neurol. 2018;14(10):577–89. 30171200 10.1038/s41582-018-0058-z

[26] Paris A, Bora P, Parolo S, MacCannell D, Monine M, van der Munnik N, *et al*. A pediatric quantitative systems pharmacology model of neurofilament trafficking in spinal muscular atrophy treated with the antisense oligonucleotide nusinersen. CPT: Pharmcomet Syst Pharmacol. 2023;12(2):196–206. 10.1002/psp4.12890 PMC9931427 36471456

[27] Paris A, Bora P, Parolo S, Monine M, Tong X, Eraly S,* et al*. An age-dependent mathematical model of neurofilament trafficking in healthy conditions. CPT Pharmacomet Syst Pharmacol. 2022;11(4):447–57. 10.1002/psp4.12770 PMC9007607 35146969

[28] Machacek M, Garcia-Montoya E, McColgan P, Sanwald-Ducray P, Mazer NA. NfL concentration in CSF is a quantitative marker of the rate of neurodegeneration in aging and Huntington's disease: a semi-mechanistic model-based analysis. 2024;18. 10.3389/fnins.2024.1420198 10.3389/fnins.2024.1420198 PMC11253127 39022122

[29] Azizi S, Hier DB, Allen B, Obafemi-Ajayi T, Olbricht GR, Thimgan MS, *et al*. A kinetic model for blood biomarker levels after mild traumatic brain injury. Front Neurol. 2021;12:668606. 34295300 10.3389/fneur.2021.668606 PMC8289906

[30] Velasco R, Marco C, Domingo-Domenech E, Stradella A, Santos C, Laquente B, *et al*. Plasma neurofilament light chain levels in chemotherapy-induced peripheral neurotoxicity according to type of anticancer drug. Eur J Neurol. 2024. 10.1111/ene.16369. 38952074 10.1111/ene.16369 PMC11295167

[31] Henningsson A SA, Loos WJ, Verweij J, Silvander M, Karlsson MO. Population Pharmacokinetic Model for Cremophor EL. PAGE. 2005;https://www.page-meeting.org/?abstract=770. Accessed 01 May 2022

[32] Fu Q, Sun X, Lustburg MB, Sparreboom A, Hu S. Predicting paclitaxel disposition in humans with whole-body physiologically-based pharmacokinetic modeling. CPT Pharmacomet Syst Pharmacol. 2019;8(12):931–9. 10.1002/psp4.12472 PMC6930855 31671477

[33] Mahmood I. Theoretical versus empirical allometry: facts behind theories application to pharmacokinetics. J Pharm Sci. 2010;99(7):2927–33. 20127826 10.1002/jps.22073

[34] Fridén M, Gupta A, Antonsson M, Bredberg U, Hammarlund-Udenaes M. In vitro methods for estimating unbound drug concentrations in the brain interstitial and intracellular fluids. Drug Metab Dispos. 2007;35(9):1711–9. 17591680 10.1124/dmd.107.015222

[35] Kuh HJ, Jang SH, Wientjes MG, Au JL. Computational model of intracellular pharmacokinetics of paclitaxel. J Pharmacol Exp Ther. 2000;293(3):761–70. 10869374

[36] Sjölin K, Kultima K, Larsson A, Freyhult E, Zjukovskaja C, Alkass K, *et al*. Distribution of five clinically important neuroglial proteins in the human brain. Mol Brain. 2022;15(1): 52. 35765081 10.1186/s13041-022-00935-6 PMC9241296

[37] Yuan A, Sasaki T, Rao MV, Kumar A, Kanumuri V, Dunlop DS,* et al*. Neurofilaments form a highly stable stationary cytoskeleton after reaching a critical level in axons. J Neurosci. 2009;29(36):11316–29. 19741138 10.1523/JNEUROSCI.1942-09.2009 PMC2788791

[38] Keddie S, Smyth D, Keh RYS, Chou MKL, Grant D, Surana S,* et al*. Peripherin is a biomarker of axonal damage in peripheral nervous system disease. Brain. 2023;146(11):4562–73. 37435933 10.1093/brain/awad234 PMC10629771

[39] Sparreboom A, van Zuylen L, Brouwer E, Loos WJ, de Bruijn P, Gelderblom H, *et al.* Cremophor EL-mediated Alteration of Paclitaxel Distribution in Human Blood: Clinical Pharmacokinetic Implications. Can Res. 1999;59(7):1454–7. 10197613

[40] Greenberger LM, Lothstein L, Williams SS, Horwitz SB. Distinct P-glycoprotein precursors are overproduced in independently isolated drug-resistant cell lines. Proc Natl Acad Sci U S A. 1988;85(11):3762–6. 2897689 10.1073/pnas.85.11.3762 PMC280298

[41] Svoboda M, Wlcek K, Taferner B, Hering S, Stieger B, Tong D,* et al.* Expression of organic anion-transporting polypeptides 1B1 and 1B3 in ovarian cancer cells: relevance for paclitaxel transport. Biomed Pharmacother. 2011;65(6):417–26. 10.1016/j.biopha.2011.04.031 21719246

[42] Staff NP, Fehrenbacher JC, Caillaud M, Damaj MI, Segal RA, Rieger S. Pathogenesis of paclitaxel-induced peripheral neuropathy: a current review of in vitro and in vivo findings using rodent and human model systems. Exp Neurol. 2020;324:113121. 31758983 10.1016/j.expneurol.2019.113121 PMC6993945

[43] Gelderblom H, Verweij J, Nooter K, Sparreboom A. Cremophor EL: the drawbacks and advantages of vehicle selection for drug formulation. Eur J Cancer (Oxford, England : 1990). 2001;37(13):1590–8. 10.1016/s0959-8049(01)00171-x 11527683

[44] Patai R, Kiss T, Gulej R, Nyul-Toth A, Csik B, Chandragiri SS, *et al*. Transcriptomic profiling of senescence effects on blood-brain barrier-related gene expression in brain capillary endothelial cells in a mouse model of paclitaxel-induced chemobrain. Geroscience. 2025;47(3):3677–91. 39976844 10.1007/s11357-025-01561-5 PMC12181502

[45] Yuan A, Sasaki T, Kumar A, Peterhoff CM, Rao MV, Liem RK, *et al*. Peripherin is a subunit of peripheral nerve neurofilaments: implications for differential vulnerability of CNS and peripheral nervous system axons. J Neurosci. 2012;32(25):8501–8. 22723690 10.1523/JNEUROSCI.1081-12.2012 PMC3405552

[46] Paxinos G, Watson C. The rat brain in stereotaxic coordinates. Academic Press; 2013.

[47] Syková E, Nicholson C. Diffusion in brain extracellular space. Physiol Rev. 2008;88(4):1277–340. 18923183 10.1152/physrev.00027.2007 PMC2785730

[48] Nicholson C, Syková E. Extracellular space structure revealed by diffusion analysis. Trends Neurosci. 1998;21(5):207–15. 9610885 10.1016/s0166-2236(98)01261-2

[49] Forbes RM, Cooper AR, Mitchell HH. The composition of the adult human body as determined by chemical analysis. J Biol Chem. 1953;203(1):359–66. 13069519

[50] Everett NB, Simmons B, Lasher EP. Distribution of blood (Fe 59) and plasma (I 131) volumes of rats determined by liquid nitrogen freezing. Circ Res. 1956;4(4):419–24. 13330185 10.1161/01.res.4.4.419

[51] Bass NH, Lundborg P. Postnatal development of bulk flow in the cerebrospinal fluid system of the albino rat: clearance of carboxyl-( 14 C)inulin after intrathecal infusion. Brain Res. 1973;52:323–32. 4739806 10.1016/0006-8993(73)90668-9

