# Supplementary material for: Evaluation of sNfL as a Biomarker for Paclitaxel-Induced Peripheral Neurotoxicity Through an Integrated PKPD Model
Source: Pharm Res. 2026 Mar 13;43(4):1071–88. doi: 10.1007/s11095-026-04053-z (PMC13179264; doi:10.1007/s11095-026-04053-z)
Supplement: Supplementary file 1 — (PDF 124 KB) [file 11095_2026_4053_MOESM1_ESM.pdf]

```
$PROBLEM   PTX PK-NfL model

;; PTX PK-NfL model


$INPUT      ID OID=DROP TIME EVID DV=DROP DVID AMT RATE DUR GDOSE WT GENDER ROA VEH SRC SPC DV CMT DOSPTX SIM

$DATA       PTX_PK_NFL_dataset.csv

            IGNORE=@ IGNORE(DVID.EQ.9) IGNORE(DVID.EQ.10) IGNORE(DVID.EQ.8) IGNORE(ID.EQ.24012) IGNORE(TIME.EQ.70) IGNORE(TIME.EQ.237)


$SUBROUTINE ADVAN14 TOL=12


$MODEL      COMP=(PTX_C)                    ;Compartment 1 describing unbound PTX concentration in central compartment

            COMP=(PTX_P1)                   ;Compartment 2 describing unbound PTX concentration in peripheral compartment

            COMP=(CRE_C)                    ;Compartment 3 describing unbound CrEL concentration in central compartment

            COMP=(CRE_P1)                   ;Compartment 4 describing unbound CrEL concentration in peripheral compartment 1

            COMP=(CRE_P2)                   ;Compartment 5 describing unbound CrEL concentration in peripheral compartment 2

            COMP=(AUC)                      ;Compartment 6 describing total PTX concentration in central compartment

            COMP=(DRG_EXTRA)                ;Compartment 7 describing unbound PTX concentration in dorsal root ganglia (DRG) extracellular compartment

            COMP=(SN_EXTRA)                 ;Compartment 8 describing unbound PTX concentration in sciatic nerve (SN) extracellular compartment, representing peripheral nerves

            COMP=(BRN_EXTRA)                ;Compartment 9 describing unbound PTX concentration in brain extracellular compartment

            COMP=(BRN_PERIEXTRA)            ;Compartment 10 describing unbound PTX concentration in secondary brain compartment

            COMP=(NFL_SERUM)                ;Compartment 11 describing NfL concentration in serum

            COMP=(NFL_CSF)                  ;Compartment 12 describing NfL concentration in cerebrospinal fluid (CSF)
```

COMP=(NFL_BRAIN) ;Compartment 13 describing NfL concentration in brain

COMP=(NFL_PNS) ;Compartment 14 describing NfL concentration in peripheral nerves and DRG combined

$PK

;;----- PTX PK parameters (plasma) --------------

CLP = THETA(1)*((WT/0.300)**0.75) ;L/h allometric scalling of a plasma clearance, 0.300 kg is assumed to be median weight of the rat

V1P = THETA(2)*((WT/0.300)**1) ;L allometric scalling of a volume of distribution of central compartment, 0.300 kg is assumed to be median weight of the rat

Q2P = THETA(3)*((WT/0.300)**0.75) ;L/h allometric scalling of a intercompartmental clearance, 0.300 kg is assumed to be median weight of the rat

V2P = THETA(4)*((WT/0.300)**1) ;L allometric scalling of a volume of distribution of peripheral compartment, 0.300 kg is assumed to be median weight of the rat

TMAX = THETA(5) ;mg/h maximum transport capacity of PTX to the peripheral compartment

KMT = (THETA(6)/1000) ;ug/L concentration at which the transport rate is half-maximal

K10P = CLP/V1P ;/h

K12P = Q2P/V1P ;/h

K21P = Q2P/V2P ;/h

;;----- CrEL PK parameters (plasma) --------------

;;----- Based on Henningsson A SA, Loos WJ, Verweij J, Silvander M, Karlsson MO. Population Pharmacokinetic Model for Cremophor EL. PAGE. 2005;https://www.page-meeting.org/?abstract=770

V1C = 4.54*((WT/70)**1) ;L allometric scalling of a volume of distribution of central compartment, 70 kg is assumed to be median weight of the human

V2C = 1.32*((WT/70)**1) ;L allometric scalling of a volume of distribution of peripheral compartment 1, 70 kg is assumed to be median weight of the human

V3C = 3.53*((WT/70)**1) ;L allometric scalling of a volume of distribution of peripheral compartment 2, 70 kg is assumed to be median weight of the human

Q12 = 1.17*((WT/70)**0.75) ;L/h allometric scalling of intercompartmental clearance 1, 70 kg is assumed to be median weight of the human

Q13 = 0.479*((WT/70)**0.75) ;L/h allometric scalling of intercompartmental clearance 2, 70 kg is assumed to be median weight of the human

VMC = THETA(8) ;mL/h maximum elimination capacity of CrEL

KMC = 2.57 ;mL/L concentration at which the elimination rate is half-maximal

K12C = Q12/V1C ;/h

K21C = Q12/V2C ;/h

K13C = Q13/V1C ;/h

K31C = Q13/V3C ;/h

BCREL = 4.46 ;unitless slope of binding of PTX to the predicted concentrations of CrEL

;;----- PTX PK parameters (DRG) ----------------

KP_UU_DRG = 4.25 ; CLin,BDB/CLout,BDB

KP_UU_CELL_DRG = 1.45 ; CLin,CB/CLout,CB

CLOUT_BDB_DRG = (THETA(9)/1E6)*((WT/0.300)**0.75) ;L/h allometric scalling of a PTX clearance from DRG, 0.300 kg is assumed to be median weight of the rat

WT_DRG = ((40*WT)/0.300)/1E6 ;L DRG weight, assumed to be 40 mg for 0.300 kg rat. The tissue density is assumed to be 1 mL/g

VIN_DRG = (0.8/1E3)*WT_DRG*1E3 ;L volume of intracellular fluid in DRG. The volume of intracellular fluid in DRG is assumed to be 0.8 mL/g DRG

VEX_DRG = (0.2/1E3)*WT_DRG*1E3 ;L volume of extracellular fluid in DRG. The volume of extracellular fluid in DRG is assumed to be 0.2 mL/g DRG

FU = 0.069 ;unitless unbound fraction of PTX in plasma in the absence of CrEL

CLIN_BDB_DRG = KP_UU_DRG*CLOUT_BDB_DRG

KIN_BDB_DRG = CLIN_BDB_DRG/V1P

KOUT_BDB_DRG = CLOUT_BDB_DRG/VEX_DRG

BMAX_DRG = THETA(10) ;mg/L initial maximum PTX-binding capacity in DRG cells

KD = THETA(11)*1E-3 ;ug/L dissociation constant for PTX binding to saturable tubulin binding sites in DRG cells

FPTX_DRG = THETA(12) ;unitless unbound fraction of PTX in DRG

;;----- PTX PK parameters (SN) -----------------

KP_UU_SN = 4.48 ; CLin,BNB/CLout,BNB

KP_UU_CELL_SN = 0.55 ; CLin,CB/CLout,CB

CLOUT_BNB_SN = (THETA(13)/1E6)*((WT/0.300)**0.75) ;L/h allometric scalling of a PTX clearance from SN, 0.300 kg is assumed to be median weight of the rat

WT_SN = (((2.781*1000)*WT)/0.300)/1E6 ;L SN weight, assumed to be 2.781 g for 0.300 kg rat. The tissue density is assumed to be 1 mL/g

VIN_SN = (0.5/1E3)*WT_SN*1E3 ;L
volume of intracellular fluid in SN. The volume of intracellular fluid in SN is assumed to be 0.5 mL/g SN

VEX_SN = (0.5/1E3)*WT_SN*1E3 ;L
volume of extracellular fluid in SN. The volume of extracellular fluid in SN is assumed to be 0.5 mL/g SN

CLIN_BNB_SN = KP_UU_SN*CLOUT_BNB_SN

KIN_BNB_SN = CLIN_BNB_SN/V1P

KOUT_BNB_SN = CLOUT_BNB_SN/VEX_SN

BMAX_SN = THETA(14) ;mg/L
initial maximum PTX-binding capacity in SN cells

KD_SN = KD
;ug/L dissociation constant for PTX binding to saturable tubulin binding sites in SN cells, assumed to be the same as in DRG

FPTX_SN = FPTX_DRG
;unitless unbound fraction of PTX in SN, to be the same as in SN

;;----- PTX PK parameters (BRN) -----------------

KP_UU_BRN = 0.032 ; CLin,BBB/CLout,BBB

KP_UU_CELL_BRN = 11.2 ; CLin,CB/CLout,CB

CLOUT_BBB_BRN = (THETA(16)/1E6)*((WT/0.300)**0.75) ;L/h
allometric scalling of a PTX clearance from brain, 0.300 kg is assumed to be median weight of the rat

WT_BRN = ((1.8*1E3*WT)/0.300)/1E6 ;L
Brain weight, assumed to be 1.8 g for rat and 0.4 g for mouse. The tissue density is assumed to be 1 mL/g

IF(SRC.EQ.4) WT_BRN = (0.4*1E3)/1E6

IF(SRC.EQ.6) WT_BRN = (0.4*1E3)/1E6

VIN_BRN = (0.8/1E3)*WT_BRN*1E3 ;L
volume of intracellular fluid in brain. The volume of intracellular fluid in brain is assumed to be 0.8 mL/g brain

VEX_BRN = (0.2/1E3)*WT_BRN*1E3 ;L
volume of extracellular fluid in brain. The volume of extracellular fluid in brain is assumed to be 0.8 mL/g brain

CLIN_BBB_BRN = KP_UU_BRN*CLOUT_BBB_BRN

KIN_BBB_BRN = CLIN_BBB_BRN/V1P

KOUT_BBB_BRN = CLOUT_BBB_BRN/VEX_BRN

Q_BRN = (THETA(17)/1E6)*((WT/0.300)**0.75) ;L/h
allometric scalling of a intercompartmental clearance inside the brain, 0.300 kg is assumed to be median weight of the rat

VP_BRN = (THETA(18)/1E3)*((WT/0.300)**1) ;L/h allometric scalling of a volume of distribution of second compartment in the brain brain, 0.300 kg is assumed to be median weight of the rat

K9_10 = Q_BRN/VEX_BRN

K10_9 = Q_BRN/VP_BRN

BMAX_BRN = THETA(19)
;mg/L initial maximum PTX-binding capacity in brain cells

KD_BRN = 0.004208248 ;mg/L
dissociation constant for PTX binding to saturable tubulin binding sites in brain cells

FPTX_BRN = FPTX_DRG
;unitless unbound fraction of PTX in brain, to be the same as in brain

;-------------NFL PD PLASMA------------------

```
BASE_SERUM_NFL = THETA(27)*EXP(ETA(1))
        ;pg/mL      NfL concentration in serum

BASE_CSF_NFL   = THETA(28)*EXP(ETA(2))                          ;pg/mL
        NfL concentration in CSF

BASE_BRN_NFL   = THETA(29)*1E6
        ;pg/mL      NfL concentration in brain

BASE_PN_NFL    = THETA(30)*1E6
        ;pg/mL sum of NfL concentration in SN and DRG


AMT_BASE_BRN_NFL   = BASE_BRN_NFL*WT_BRN*1E3                    ;pg
        NfL amount in brain

AMT_BASE_PN_NFL    = BASE_PN_NFL*(WT_SN+WT_DRG)*1E3
        ;pg         NfL amount in SN and DRG

AMT_BASE_SERUM_NFL = BASE_SERUM_NFL*5
        ;pg         NfL amount in serum, the volume of serum is assumed to be 5 mL

AMT_BASE_CSF_NFL   = BASE_CSF_NFL*0.25
        ;pg         NfL amount in CSF, the volume of CSF is assumed to be 0.25 mL


KOUT_PLASMA   = (10.1/24)*((WT/70)**(-0.25))
        ;/h first-order elimination rate constant of NfL from serum

KOUT_CSF      = KOUT_PLASMA*AMT_BASE_SERUM_NFL/AMT_BASE_CSF_NFL

KIN_CSF       = AMT_BASE_CSF_NFL*KOUT_CSF


EMAX_PTX_BRN   = THETA(32)*EXP(ETA(4))*1E9

EMAX_PTX_PNS   = EMAX_PTX_BRN


A_0(11) = AMT_BASE_SERUM_NFL

A_0(12) = AMT_BASE_CSF_NFL

A_0(13) = AMT_BASE_BRN_NFL

A_0(14) = AMT_BASE_PN_NFL


$DES


 ;------------non linear PTX distribution------------
```

```
Q_NL   = (TMAX/(KMT + (A(1)/V1P)))*((WT/0.300)**0.75)                          ;L/h non linear intercompartmental clearance of PTX, 0.300 kg is assumed to be median weight of the rat

KQ_NL  = Q_NL/V1P


;-------------non linear Cre elimination-------------

CL_NL  = (VMC/(KMC + (A(3)/V1C)))*((WT/0.300)**0.75)        ;L/h non linear clearance of CrEL, 0.300 kg is assumed to be median weight of the rat

KCL_NL = CL_NL/V1C



;-------------Differential equations for PTX plasma PK-------------

DADT(1)  = -K10P*A(1) - KQ_NL*A(1) -K12P*A(1) +K21P*A(2)

DADT(2)  = -K21P*A(2) + KQ_NL*A(1) +K12P*A(1)


;-------------Differential equations for CrEL plasma PK-------------

DADT(3)  = -KCL_NL*A(3) -K12C*A(3) -K13C*A(3) +K31C*A(5)+K21C*A(4)

DADT(4)  = -K21C*A(4) + K12C*A(3)

DADT(5)  = -K31C*A(5) + K13C*A(3)


;-------------Differential equations for PTX AUC determination-------------

CPTOTAL  = (A(1)/V1P)*(1+ BCREL*(A(3)/V1C))
                    ;mg/L total PTX concentration

DADT(6)  = CPTOTAL


XRCAUC  = A(6)

XRCAUCU = XRCAUC*1000
                              ;ng · hr/mL PTX AUC


PTXCONC = A(1)/V1P
                              ;mg/L unbound paclitaxel concentration in central compartment

CRECONC = A(3)/V1C
                              ;mg/L unbound CrEL concentration in central compartment
```

;-------------Differential equations for PTX PK in DRG-------------

DADT(7) = KIN_BDB_DRG*((A(1)*FU) + (BCREL*CRECONC*PTXCONC*V1P*FPTX_DRG)) - KOUT_BDB_DRG*A(7) ;mg unbound PTX amount distribution across blood-DRG barrier

XDRGU_EX = A(7) ;mg unbound PTX amount in extracellular space in DRG

XDRGU_EX_CONC = A(7)/VEX_DRG ;mg/L unbound PTX concentration in extracellular space in DRG

XDRGU_IN_CONC = XDRGU_EX_CONC*KP_UU_CELL_DRG ;mg/L unbound PTX concentration distribution in intracellular space in DRG

XTBMAX_DRG = BMAX_DRG*(1+((THETA(20)*(XRCAUC**THETA(21)))/((THETA(22)**THETA(21))+(XRCAUC**THETA(21))))) ;mg/L maximum PTX-binding capacity in DRG cells, rep. cum. AUC as cov

XDRGB_IN_CONC = (XTBMAX_DRG*XDRGU_IN_CONC)/(KD+XDRGU_IN_CONC) ;mg/L bound PTX to tubulin concentration in intracellular space in DRG

XDRGU_IN = XDRGU_IN_CONC*VIN_DRG ;mg unbound PTX amount in intracellular space in DRG

XDRGB_IN = XDRGB_IN_CONC*VIN_DRG ;mg bound PTX to tubulin amount in intracellular space in DRG

XDRGTOT = XDRGU_EX + (XDRGU_IN_CONC + XDRGB_IN_CONC)*VIN_DRG ;mg total PTX amount in intracellular space in DRG

```
;-------------Differential equations for PTX PK in SN-------------

DADT(8)  =  KIN_BNB_SN*((A(1)*FU) +
(BCREL*CRECONC*PTXCONC*V1P*FPTX_SN)) - KOUT_BNB_SN*A(8)
                              ;mg          unbound PTX amount distribution across blood-
nerve barrier

XSNU_EX      = A(8)

              ;mg          unbound PTX amount in extracellular space in SN
XSNU_EX_CONC  = A(8)/VEX_SN

              ;mg/L        unbound PTX concentration in extracellular space in SN

XSNU_IN_CONC  = XSNU_EX_CONC*KP_UU_CELL_SN

              ;mg/L        unbound PTX concentration distribution in intracellular space in
SN
XTBMAX_SN    = BMAX_SN*EXP(XRCAUC*THETA(26))

              ;mg/L        maximum PTX-binding capacity in SN cells, rep. cum. AUC as
cov
XSNB_IN_CONC  = (XTBMAX_SN*XSNU_IN_CONC)/(KD_SN+XSNU_IN_CONC)

              ;mg/L        bound PTX to tubulin concentration in intracellular space in SN

XSNU_IN      = XSNU_IN_CONC*VIN_SN

              ;mg          unbound PTX amount in intracellular space in SN
XSNB_IN      = XSNB_IN_CONC*VIN_SN

              ;mg          bound PTX to tubulin amount in intracellular space in SN

XSNTOT       = XSNU_EX + (XSNU_IN_CONC + XSNB_IN_CONC)*VIN_SN

              ;mg          total PTX amount in intracellular space in SN

;-------------Differential equations for PTX PK in brain-------------
```

DADT(9) = KIN_BBB_BRN*((A(1)*FU) + (BCREL*CRECONC*PTXCONC*V1P*FPTX_BRN)) - KOUT_BBB_BRN*A(9) - K9_10* A(9) + K10_9* A(10) ;mg unbound PTX amount distribution across blood-brain barrier

DADT(10) = K9_10* A(9) - K10_9* A(10) ;mg unbound PTX amount distribution to the second compartment in the brain

XBRNU_EX = A(9) ;mg unbound PTX amount in extracellular space in brain

XBRNU_EX_CONC = A(9)/VEX_BRN ;mg/L unbound PTX concentration in extracellular space in brain

XBRNU_IN_CONC = XBRNU_EX_CONC*KP_UU_CELL_BRN ;mg/L unbound PTX concentration distribution in intracellular space in brain

XTBMAX_BRN = BMAX_BRN*(1+((THETA(20)*(XRCAUC**THETA(21)))/((THETA(22)**THETA(21))+(XRCAUC**THETA(21))))) ;mg/L maximum PTX-binding capacity in brain cells, rep. cum. AUC as cov

XBRNB_IN_CONC = (XTBMAX_BRN*XBRNU_IN_CONC)/(KD_BRN+XBRNU_IN_CONC) ;mg/L bound PTX to tubulin concentration in intracellular space in brain

XBRNU_IN = XBRNU_IN_CONC*VIN_BRN ;mg unbound PTX amount in intracellular space in brain

XBRNB_IN = XBRNB_IN_CONC*VIN_BRN ;mg bound PTX to tubulin amount in intracellular space in brain

XBRNTOT = XBRNU_EX + A(10) +(XBRNU_IN_CONC + XBRNB_IN_CONC)*VIN_BRN ; total in mg ;mg total PTX amount in intracellular space in brain

```
;-------------Differential equations for NfL kinetics-------------

TOTB_IN_CONC_BRN = XBRNB_IN
PTX_EFF_BRN      = EMAX_PTX_BRN*TOTB_IN_CONC_BRN

                ;            bound PTX to tubulin effect on NfL leakage from brain


TOTB_IN_CONC_PN = XDRGB_IN + XSNB_IN
PTX_EFF_PNS     = EMAX_PTX_PNS*TOTB_IN_CONC_PN

                ;            bound PTX to tubulin effect on NfL leakage from nerves and
DRG combined

DADT(11) =   KOUT_CSF*A(12) + PTX_EFF_PNS - KOUT_PLASMA*A(11)
                                                                        ;pg
                NfL amount distribution in serum compartment
DADT(12) =   PTX_EFF_BRN + KIN_CSF - KOUT_CSF*A(12)
                                                                        ;pg
                NfL amount distribution in CSF compartment
DADT(13) = - PTX_EFF_BRN

                ;pg          NfL amount distribution in brain compartment
DADT(14) = - PTX_EFF_PNS

                ;pg          NfL amount distribution in nerves and DRG combined
compartment

$ERROR

  PTX_CONC = A(1)/V1P

                ;mg/L        unbound PTX concentration in central compartment
  CRE_CONC = A(3)/V1C

                ;mg/L        unbound CrEL concentration in central compartment
```

CPTOT = PTX_CONC*(1+ BCREL*CRE_CONC)

;mg/L total PTX concentration in central compartment

CPT = CPTOT*1000

;ng/mL total PTX concentration in central compartment

RCAUC = A(6)

;mg · hr/L rep. cAUC

RCAUCU = RCAUC*1000

;ng · hr/mL rep. cAUC

DRGU_EX = A(7)

;mg unbound PTX amount in extracellular space in DRG

DRGU_EX_CONC = A(7)/VEX_DRG

;mg/L unbound PTX concentration in extracellular space in DRG

DRGU_IN_CONC = DRGU_EX_CONC*KP_UU_CELL_DRG

;mg/L unbound PTX concentration distribution in intracellular space in DRG

TBMAX_DRG = BMAX_DRG*(1+((THETA(20)*(RCAUC**THETA(21)))/((THETA(22)**THETA(21))+(RCAUC**THETA(21))))) ;mg/L maximum PTX-binding capacity in DRG cells, rep. cum. AUC as cov

DRGB_IN_CONC = (TBMAX_DRG*DRGU_IN_CONC)/(KD+DRGU_IN_CONC)

;mg/L bound PTX to tubulin concentration in intracellular space in DRG

DRGU_IN = DRGU_IN_CONC*VIN_DRG

;mg unbound PTX amount in intracellular space in DRG

DRGB_IN = DRGB_IN_CONC*VIN_DRG

;mg bound PTX to tubulin amount in intracellular space in DRG

DRGTOT = DRGU_EX + (DRGU_IN_CONC + DRGB_IN_CONC)*VIN_DRG ;mg total PTX amount in DRG

DRGTOT_CONC = DRGTOT/WT_DRG

;mg/L total PTX concentration in DRG

DRGT = DRGTOT_CONC*1000 ;ng/mL total PTX concentration in DRG

SNU_EX = A(8)

;mg unbound PTX amount in extracellular space in SN

SNU_EX_CONC = A(8)/VEX_SN

;mg/L unbound PTX concentration in extracellular space in SN

SNU_IN_CONC = SNU_EX_CONC*KP_UU_CELL_SN

;mg/L unbound PTX concentration distribution in intracellular space in SN

TBMAX_SN = BMAX_SN*EXP(RCAUC*THETA(26))

;mg/L maximum PTX-binding capacity in SN cells, rep. cum. AUC as cov

SNB_IN_CONC = (TBMAX_SN*SNU_IN_CONC)/(KD_SN+SNU_IN_CONC)

;mg/L bound PTX to tubulin concentration in intracellular space in SN

SNU_IN = SNU_IN_CONC*VIN_SN

;mg unbound PTX amount in intracellular space in SN

SNB_IN = SNB_IN_CONC*VIN_SN

;mg bound PTX to tubulin amount in intracellular space in SN

SNTOT = SNU_EX + (SNU_IN_CONC + SNB_IN_CONC)*VIN_SN ;mg total PTX amount in SN

SNTOT_CONC = SNTOT/WT_SN

;mg/L total PTX concentration in SN

SNT = SNTOT_CONC*1000 ;ng/mL total PTX concentration in SN

BRNU_EX = A(9)

;mg unbound PTX amount in extracellular space in brain

BRNU_EX_CONC = A(9)/VEX_BRN

;mg/L unbound PTX concentration in extracellular space in brain

BRNU_IN_CONC = BRNU_EX_CONC*KP_UU_CELL_BRN

;mg/L unbound PTX concentration distribution in intracellular space in brain

TBMAX_BRN = BMAX_BRN*(1+((THETA(20)*(RCAUC**THETA(21)))/((THETA(22)**THETA(21))+(RCAUC**THETA(21))))) ;mg/L maximum PTX-binding capacity in brain cells, rep. cum. AUC as cov

BRNB_IN_CONC = (TBMAX_BRN*BRNU_IN_CONC)/(KD_BRN+BRNU_IN_CONC) ;mg/L bound PTX to tubulin concentration in intracellular space in brain

BRNU_IN = BRNU_IN_CONC*VIN_BRN

;mg unbound PTX amount in intracellular space in brain

BRNB_IN = BRNB_IN_CONC*VIN_BRN

;mg bound PTX to tubulin amount in intracellular space in brain

```
 BRNTOT        = BRNU_EX + A(10) +(BRNU_IN_CONC +
BRNB_IN_CONC)*VIN_BRN
                    ;mg              total PTX amount in brain

 BRNTOT_CONC   = BRNTOT/WT_BRN

                                    ;mg/L          total PTX concentration in brain

 BRNT          = BRNTOT_CONC*1000
                                                                                    ;ng/mL
                    total PTX concentration in brain


 DEL    = 1E-6

 IPRED  = LOG(CPT + DEL)

                    ;ng/mL         total PTX concentration in plasma

 W      =  THETA(7)


 IF(DVID.EQ.2) THEN

 IPRED  = LOG(SNT + DEL)

                    ;ng/mL         total PTX concentration in nerves

 W      = THETA(23)

 ENDIF


 IF(DVID.EQ.3) THEN

 IPRED  = LOG(DRGT + DEL)

                    ;ng/mL         total PTX concentration in DRG

 W      = THETA(24)

 ENDIF


 IF(DVID.EQ.4) THEN

 IPRED  = LOG(BRNT + DEL)

                    ;ng/mL         total PTX concentration in brain

 W      = THETA(25)
```

```
  ENDIF

  NFL_SERUM = A(11)/5
                    ;pg/mL NfL concentration in serum
  NFL_CSF   = A(12)/0.25
                                                                    ;pg/mL NfL
concentration in CSF
  NFL_BRN   = A(13)/(WT_BRN*1E3)
                                                                    ;pg/mL NfL
concentration in brain
  NFL_PNS   = A(14)/((WT_SN+WT_DRG)*1E3)
                                                                    ;pg/mL NfL
concentration in nerves and DRG combined

  IF(DVID.EQ.6) THEN
  IPRED  = LOG(NFL_SERUM + DEL)
                    ;pg/mL total NFL concentration in plasma
  W      = THETA(34)
  ENDIF

  IF(DVID.EQ.7) THEN
  IPRED  = LOG(NFL_CSF + DEL)
                                                                    ;pg/mL total
NFL concentration in CSF
  W      = THETA(35)
  ENDIF

  IRES   =  DV -  IPRED
  IF (W.EQ.0) W = 1

  IWRES  =  IRES/W
  Y      =  IPRED+W*EPS(1)
```

```
AA1  = A(1)
AA2  = A(2)
AA3  = A(3)
AA4  = A(4)
AA5  = A(5)
AA6  = A(6)
AA7  = A(7)
AA8  = A(8)
AA9  = A(9)
AA10 = A(10)
AA11 = A(11)
AA12 = A(12)
AA13 = A(13)
AA14 = A(14)

$THETA  (0,1.07323) FIX          ; 1 CLP
 (0,0.558984) FIX                ; 2 V1P
 (0,16.8238) FIX                 ; 3 Q2P
 (0,7.49171) FIX                 ; 4 V2P
 (0,0.547305) FIX                ; 5 TMAX
 (0,0.100924) FIX                ; 6 KMT

 (0,0.356814) FIX                ; 7 Res err prop
 (0,0.175047) FIX                ; 8 VMC
 (0,0.57698) FIX                 ; 9 CLOUT_BDB_DRG

 (0,0.286703) FIX                ; 10 Bmax_DRG
 (0,0.352706) FIX                ; 11 KD
 (0,0.525151,1) FIX              ; 12 FPTX_DRG
```

```
(0,772.494) FIX                         ; 13 CLOUT_BNB_SN
(0,0.144341) FIX                        ; 14 Bmax_SN
0 FIX                                             ; 15 KD_SN

(0,23.6918) FIX                         ; 16 CLOUT_BBB_BRN
(0,6.46732) FIX                         ; 17 Q_BRN
(0,3.22582) FIX                         ; 18 VP_BRN
(0,0.025915) FIX                        ; 19 Bmax_brn

(0,14.9417) FIX                         ; 20 rep.cum.AUC_EMAX_BMAX
(0,2.75525) FIX                         ; 21 rep.cum.AUC_HILL_BMAX
(0,97.9133) FIX                         ; 22 rep.cum.AUC_EC50_BMAX

(0,0.69105) FIX                         ; 23 Res err prop_sn
(0,0.493365) FIX                        ; 24 Res err prop_drg
(0,0.417205) FIX                        ; 25 Res err prop_brn
(0,0.0193666) FIX                       ; 26 Exponential_coefficient_SN

(0,17.6446)                             ; 27 BASE_PLASMA_NFL
(0,222.501)                             ; 28 BASE_CSF_NFL
(0,0.43) FIX                                      ; 29 BASE_BRN_NFL
(0,538.74) FIX                          ; 30 BASE_PN_NFL
1E-16 FIX                               ; 31 Kout-CSF

(0,0.029005)                                      ; 32 EMAX_PTX BRN=PNS
1E-16 FIX                               ; 33 EMAX_PTX PNS

(0,0.465805)                                      ; 34 Res err prop NfL serum
(0,0.371217)                                      ; 35 Res err prop NfL CSF
(0,0.291047) FIX                        ; 36 Res err prop NfL BRN
```

```
$OMEGA  0  FIX

$OMEGA  0  FIX

$OMEGA  0  FIX

$OMEGA  0  FIX

$SIGMA  1  FIX


$ESTIMATION POSTHOC MAXEVAL=9999 METHOD=1 INTER MSFO=msfb001
PRINT=1

$COVARIANCE PRINT=E MATRIX=S

$TABLE      ID TIME CMT AMT AA1 AA2 AA3 AA4 AA5 AA6 AA7 AA8 AA9 AA10
AA11 AA12 AA13 AA14 NFL_SERUM NFL_BRN NFL_CSF NFL_PNS CPTOT CPT
DVID TBMAX_DRG DRGU_EX DRGTOT DRGU_IN DRGB_IN DRGT TBMAX_SN
SNU_EX SNTOT SNU_IN SNB_IN SNT TBMAX_BRN BRNU_EX BRNTOT BRNU_IN
BRNB_IN BRNT WT SRC DOSPTX IPRED IWRES EVID CWRES CWRESI NOPRINT
ONEHEADER FILE=sdtab001

$TABLE      ID TIME V1P Q2P V2P TMAX KMT CLP NOPRINT ONEHEADER
FILE=patab001

$TABLE      ID TIME SPC VEH GENDER ROA NOPRINT ONEHEADER FILE=catab001

$TABLE      ID TIME WT DUR NOPRINT ONEHEADER FILE=cotab001
```
