# Supplementary material for: Evaluation of sNfL as a Biomarker for Paclitaxel-Induced Peripheral Neurotoxicity Through an Integrated PKPD Model
Source: Pharm Res. 2026 Mar 13;43(4):1071–88. doi: 10.1007/s11095-026-04053-z (PMC13179264; doi:10.1007/s11095-026-04053-z)
Supplement: Supplementary file 2 — (PDF 1.00 MB) [file 11095_2026_4053_MOESM2_ESM.pdf]

Evaluation of sNfL as a biomarker for paclitaxel-induced peripheral neurotoxicity through an integrated PKPD model

Eman I. K. Ibrahim[1*], Milda Girdenyté[1,2*], Yang Hu[1,3], Lorenzo Di Cesare Mannelli[4], David Balayssac[5], Jérôme Busserolles[5], Diethilde Theil[6,7], Gautier Roussignol[8], Olivier Perrault[8], Nathalie Le Berre[8], Franck Chanut[9], Mohamed Slaoui[9], Irena Loryan[1§], and Lena E. Friberg[1§]

[1]Department of Pharmacy, Uppsala University, Uppsala, Sweden

[2]Pharmacy and Pharmacology Center, Institute of Biomedical Sciences, Faculty of Medicine, Vilnius University, Vilnius, Lithuania

[3]Current affiliation: Non-Clinical Safety & DMPK, BioNTech SE, Mainz, Germany

[4]University of Florence, Department of Neuroscience, Psychology, Drug Research and Child Health – Neurofarba – Section of Pharmacology and Toxicology, Florence, Italy

[5]Université Clermont Auvergne, INSERM, U1107, NEURO-DOL, Clermont-Ferrand, France

[6] Novartis Institutes for Biomedical Research, Cambridge, MA

[7]Current affiliation: Roche Pharmaceutical Research and Early Development, Roche Innovation Center, Basel, Switzerland

[8]Sanofi, R&D, Preclinical Safety, Montpellier, France

[9]Sanofi, R&D, Preclinical Safety, Chilly Mazarin & Vitry, France

*- shared first authorship

§-shared last authorship

Corresponding authors

Irena Loryan (irena.loryan@uu.se) and Lena E. Friberg (lena.friberg@uu.se)

Supplementary materials

Table S1. Summary on the neurofilament light-chain concentration range (minimum-maximum) detected in the serum, cerebrospinal fluid (CSF), sciatic nerve (SN), dorsal root ganglia (DRG), and hippocampus at the baseline (0 hours) and after a 10 mg/kg bolus dose of CrEL-paclitaxel (72 and 240 hours).

Tissue	Units	0 hours	72 hours	240 hours
Serum	pg/mL	6.8 – 25.2	217.0 – 530.0	35.4 – 105.0
CSF	pg/mL	153.0 – 343.0	702.0 – 783.0	774.0*
SN	µg/mL	102.7 – 412.2	137.2 – 521.0	44.6 – 503.0
DRG	µg/mL	29.6 – 233.2	70.1 – 237.6	15.5 – 132.5
Hippocampus	µg/mL	0.4 – 0.5	0.7 - 0.9	0.4 – 1.0

*One data point

A)

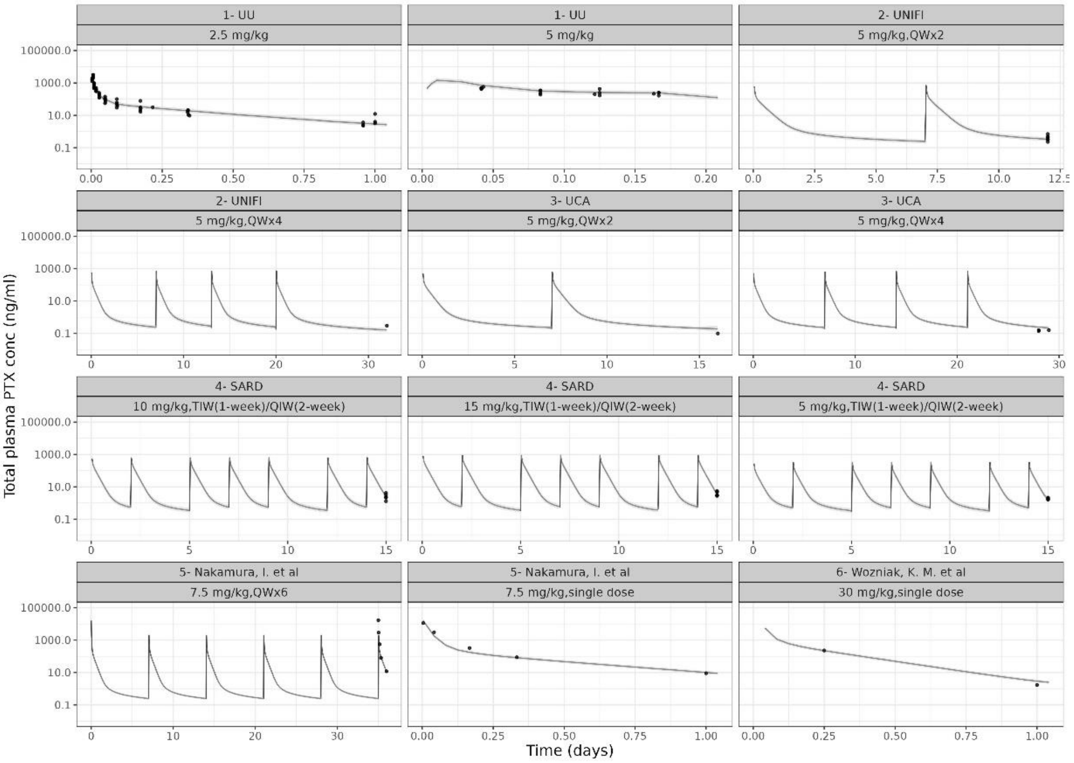
1- UU
2.5 mg/kg
1- UU
5 mg/kg
2- UNIFI
5 mg/kg,QWx2
2- UNIFI
5 mg/kg,QWx4
3- UCA
5 mg/kg,QWx2
3- UCA
5 mg/kg,QWx4
4- SARD
10 mg/kg,TIW(1-week)/QIW(2-week)
4- SARD
15 mg/kg,TIW(1-week)/QIW(2-week)
4- SARD
5 mg/kg,TIW(1-week)/QIW(2-week)
5- Nakamura, I. et al
7.5 mg/kg,QWx6
5- Nakamura, I. et al
7.5 mg/kg,single dose
6- Wozniak, K. M. et al
30 mg/kg,single dose
Total plasma PTX conc (ng/ml)
Time (days)

B)

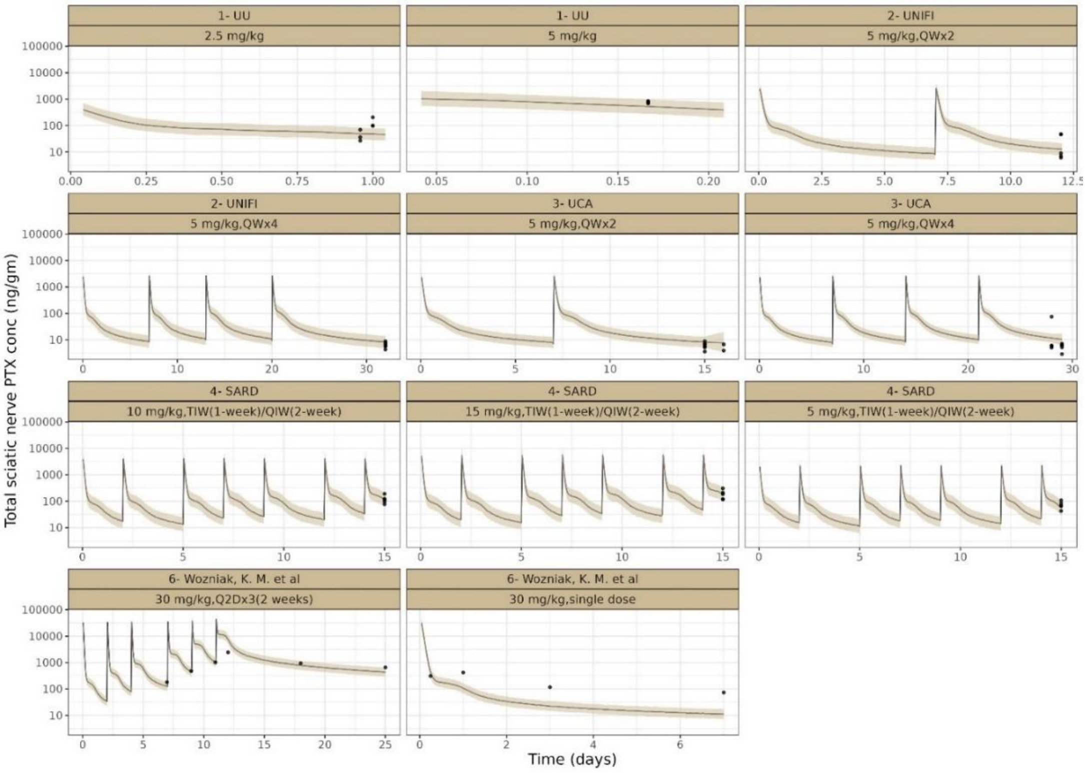
1- UU
2.5 mg/kg
1- UU
5 mg/kg
2- UNIFI
5 mg/kg,QWx2
2- UNIFI
5 mg/kg,QWx4
3- UCA
5 mg/kg,QWx2
3- UCA
5 mg/kg,QWx4
4- SARD
10 mg/kg,TIW(1-week)/QIW(2-week)
4- SARD
15 mg/kg,TIW(1-week)/QIW(2-week)
4- SARD
5 mg/kg,TIW(1-week)/QIW(2-week)
6- Wozniak, K. M. et al
30 mg/kg,Q2Dx3(2 weeks)
6- Wozniak, K. M. et al
30 mg/kg,single dose
Total sciatic nerve PTX conc (ng/gm)
Time (days)

C)

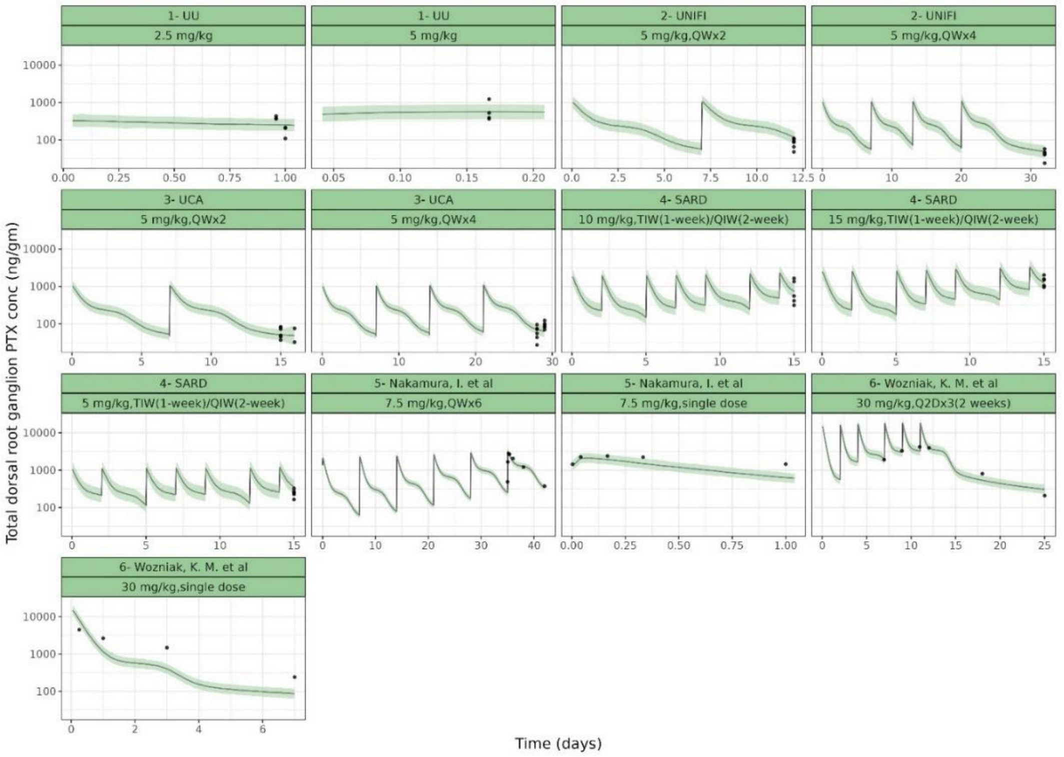
1- UU
2.5 mg/kg
1- UU
5 mg/kg
2- UNIFI
5 mg/kg,QWx2
2- UNIFI
5 mg/kg,QWx4
3- UCA
5 mg/kg,QWx2
3- UCA
5 mg/kg,QWx4
4- SARD
10 mg/kg,TIW(1-week)/QIW(2-week)
4- SARD
15 mg/kg,TIW(1-week)/QIW(2-week)
4- SARD
5 mg/kg,TIW(1-week)/QIW(2-week)
5- Nakamura, I. et al
7.5 mg/kg,QWx6
5- Nakamura, I. et al
7.5 mg/kg,single dose
6- Wozniak, K. M. et al
30 mg/kg,Q2Dx3(2 weeks)
6- Wozniak, K. M. et al
30 mg/kg,single dose
Total dorsal root ganglion PTX conc (ng/gm)
Time (days)

D)

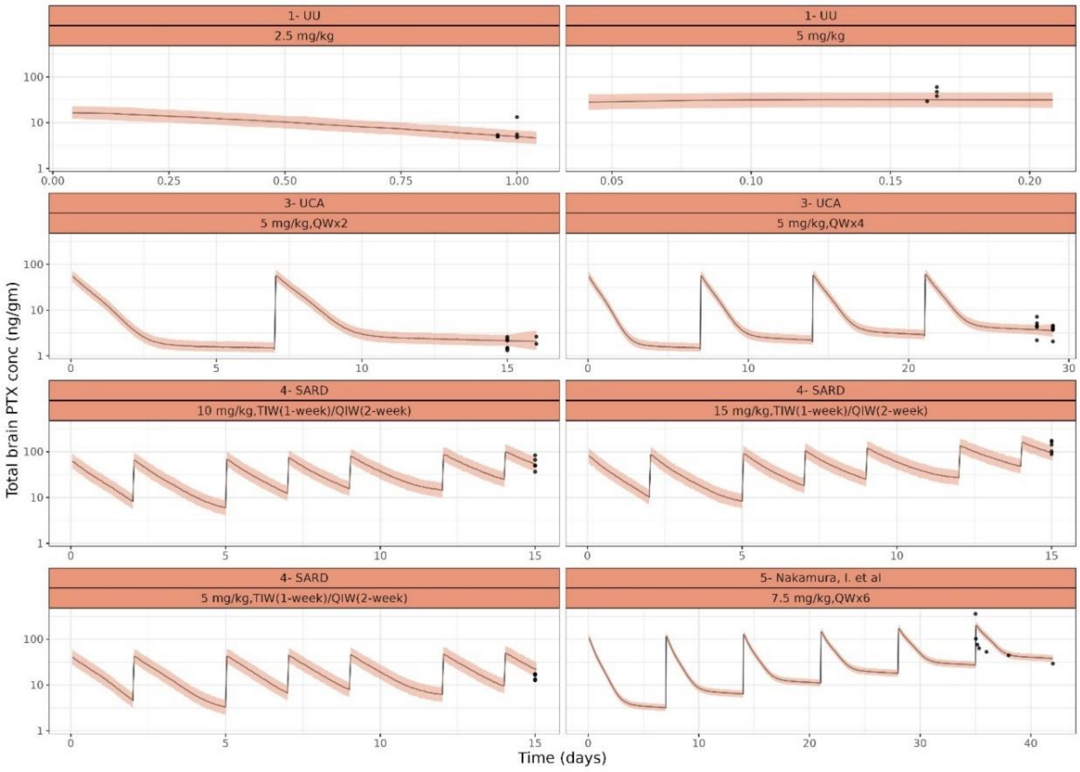
1- UU
2.5 mg/kg
1- UU
5 mg/kg
3- UCA
5 mg/kg,QWx2
3- UCA
5 mg/kg,QWx4
4- SARD
10 mg/kg,TIW(1-week)/QIW(2-week)
4- SARD
15 mg/kg,TIW(1-week)/QIW(2-week)
4- SARD
5 mg/kg,TIW(1-week)/QIW(2-week)
5- Nakamura, I. et al
7.5 mg/kg,QWx6
Total brain PTX conc (ng/gm)
Time (days)

Figure S1. The PTX plasma–PIPN sites PK model simulation-based diagnostics for the concentration-time profiles of paclitaxel (PTX) in **A)** plasma, **B)** sciatic nerve, **C)** dorsal root ganglia, and **D)** brain. The solid dots represent the observed PTX concentrations (ng/mL). Solid lines represent the medians of the model predictions. Color-shaded areas are the 90% confidence intervals of the predicted medians. Each box vertically represents the source of data: Uppsala University (UU), University of Florence (UNIFI), University Clermont Auvergne (UCA), Sanofi laboratories (SARD), and digitized data from Nakamura et al (6) and Wozniak et al (5). Abbreviations: PIPN – paclitaxel-induced peripheral neurotoxicity, QWx2 – every week for 2 weeks, QWx4 – every week for 4 weeks, TIW – three times a week, QIW – four times a week, QWx6 – every week for 6 weeks, Q2Dx3 – every 2 days for 3 doses.

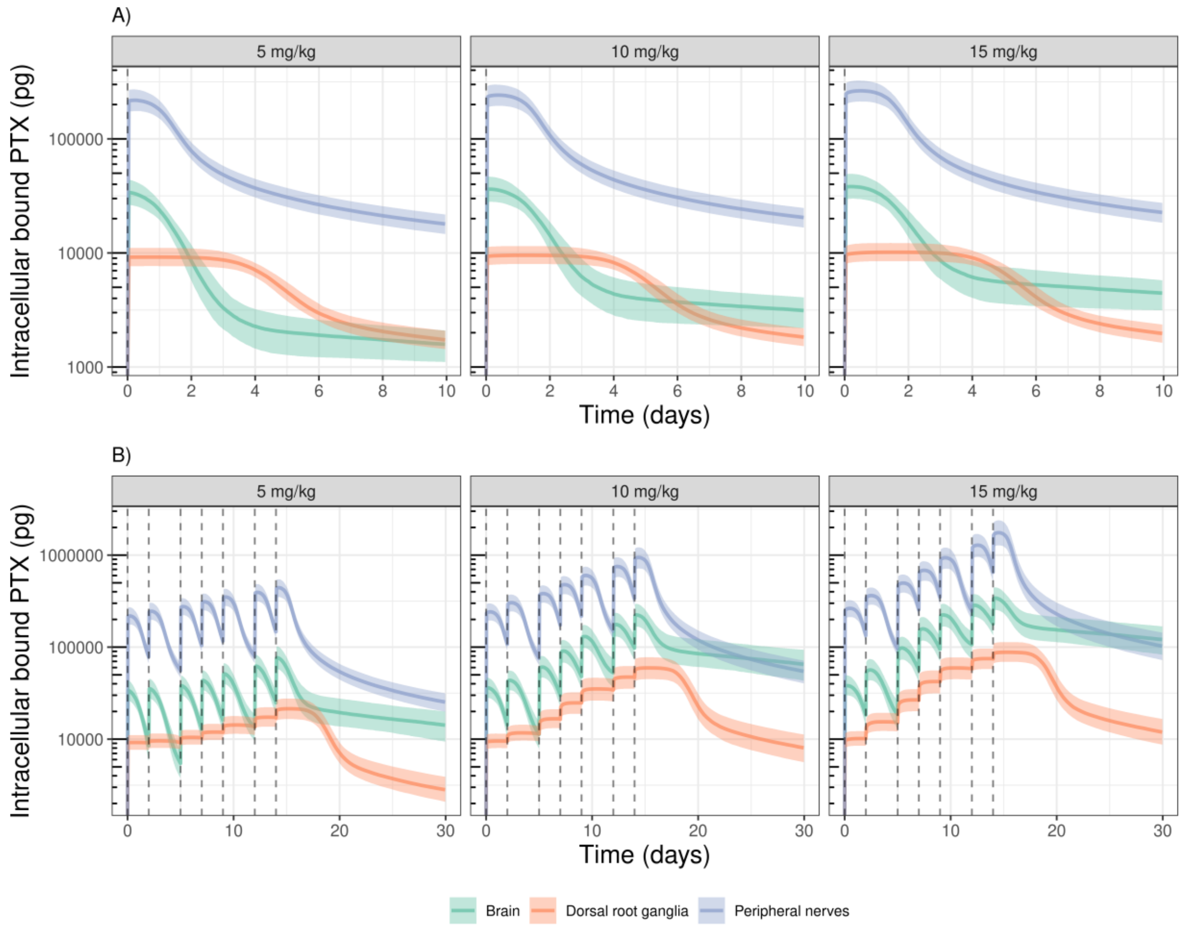

Figure S2. Simulations with parameter uncertainty from the PTX plasma–PIPN sites PK model in rats depicting the amount-time profiles of the intracellular bound paclitaxel (PTX) in the dorsal root ganglia, peripheral nerves, and brain following **A)** single dose and **B)** multiple dose (three times a week at week 1 and four times a week at week 2) administration of 5, 10 and 15 mg/kg. The solid lines represent the median, and the shaded areas are the 80 % simulation intervals. The vertical dashed lines are the PTX dosing times. PIPN – paclitaxel-induced peripheral neurotoxicity.

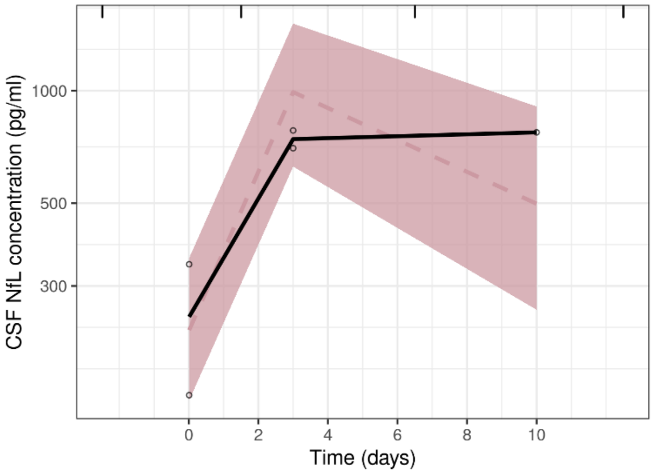

Figure S3. Visual predictive checks (VPCs) of the NfL kinetics model for the concentration-time profiles of NfL in cerebrospinal fluid (CSF). The black dots are the observed concentrations. The solid and dashed lines are the median of the observed and simulated concentrations, respectively. The color-shaded area is the 90 % confidence interval of the predicted median.

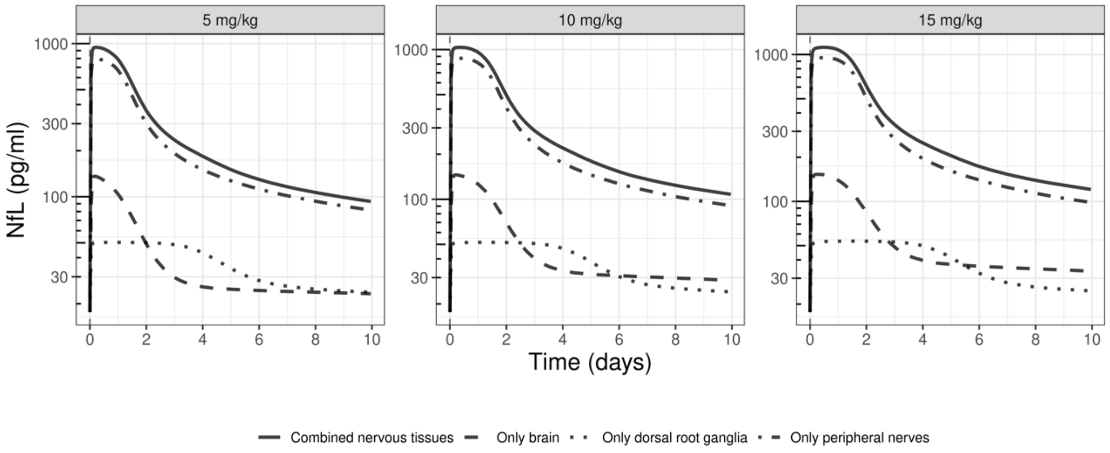

Figure S4. PTX PK- NfL model simulation-based assessment of the contributing role of PIPN sites on sNfL kinetics **A)** single dose administration of 5, 10 and 15 mg/kg. The different line types show the different nervous tissues (dashed; brain, dotted-dashed; peripheral nerves, dotted; dorsal root ganglia) contribution to the overall sNfL time-profile (solid line). The vertical dashed lines are the PTX dosing times. The solid lines represent the median. Abbreviations: PTX – paclitaxel, PK – pharmacokinetics, NfL – neurofilament light chain, PIPN – paclitaxel-induced peripheral neurotoxicity.
